# IRF5:RelA Interaction Targets Inflammatory Genes in Macrophages

**DOI:** 10.1016/j.celrep.2014.07.034

**Published:** 2014-08-21

**Authors:** David G. Saliba, Andreas Heger, Hayley L. Eames, Spyros Oikonomopoulos, Ana Teixeira, Katrina Blazek, Ariadne Androulidaki, Daniel Wong, Fui G. Goh, Miriam Weiss, Adam Byrne, Manolis Pasparakis, Jiannis Ragoussis, Irina A. Udalova

**Affiliations:** 1 Kennedy Institute of Rheumatology, University of Oxford, Roosevelt Drive, Oxford OX37FY, UK; 2 CGAT, MRC Functional Genomics Unit, University of Oxford, South Parks Road, Oxford OX13PT, UK; 3 Wellcome Trust Centre for Human Genetics, University of Oxford, Roosevelt Drive, Oxford OX3 7BN, UK; 4 Institute for Genetics, University of Cologne, Joseph-Stelzmann-Strasse 26, Cologne 50931, Germany

## Abstract

Interferon Regulatory Factor 5 (IRF5) plays a major role in setting up an inflammatory macrophage phenotype, but the molecular basis of its transcriptional activity is not fully understood. In this study, we conduct a comprehensive genome-wide analysis of IRF5 recruitment in macrophages stimulated with bacterial lipopolysaccharide and discover that IRF5 binds to regulatory elements of highly transcribed genes. Analysis of protein:DNA microarrays demonstrates that IRF5 recognizes the canonical IRF-binding (interferon-stimulated response element [ISRE]) motif in vitro. However, IRF5 binding in vivo appears to rely on its interactions with other proteins. IRF5 binds to a noncanonical composite PU.1:ISRE motif, and its recruitment is aided by RelA. Global gene expression analysis in macrophages deficient in IRF5 and RelA highlights the direct role of the RelA:IRF5 cistrome in regulation of a subset of key inflammatory genes. We map the RelA:IRF5 interaction domain and suggest that interfering with it would offer selective targeting of macrophage inflammatory activities.

## Highlights

• Genome-wide function of IRF5 in LPS-stimulated macrophages was analyzed
• IRF5 cistrome overlaps with RelA cistrome at multiple loci
• IRF5 targets regulatory elements of highly inducible inflammatory genes
• IRF5 recruitment to key inflammatory loci is assisted by RelA

## Graphical Abstract

**Figure:**
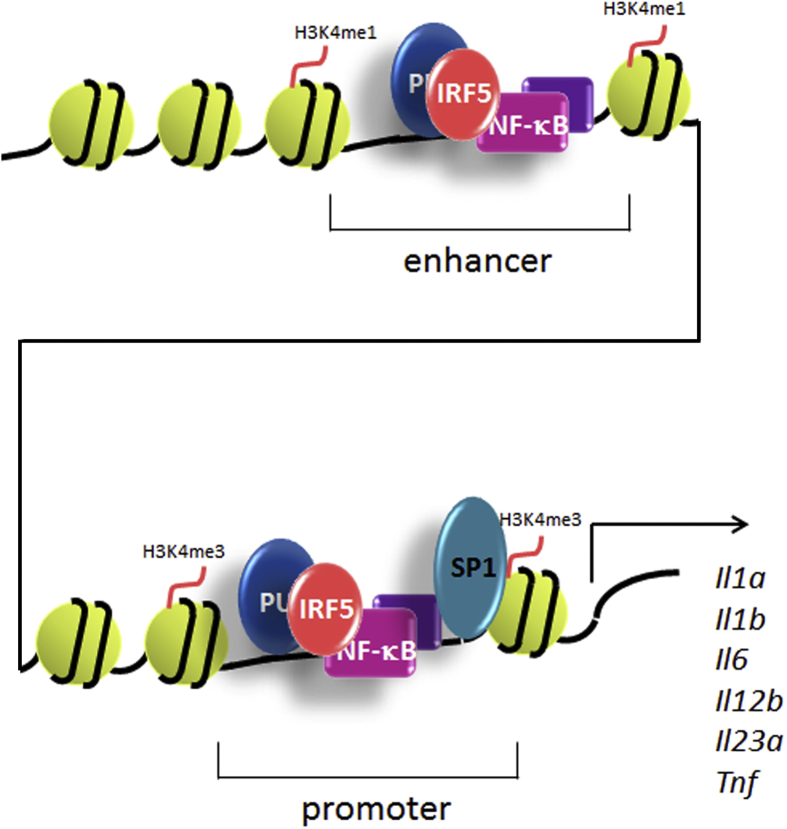

## Teaser

Saliba et al. show that the genome-wide binding of IRF5 and RelA in LPS-stimulated macrophages overlaps at multiple loci. IRF5 and RelA target regulatory elements of highly inducible inflammatory genes. IRF5 is recruited to a composite PU.1:ISRE motif, and its binding is aided by NF-κb. This study systematically defines the mechanism of IRF5 and RelA transcriptional regulation of a key subset of inflammatory genes and highlights IRF5:RelA interaction interface as a target for specific interventions.

## Introduction

A finely tuned inflammatory response to microbial and endogenous insults is essential for host survival. During inflammation, gene programs are activated by orchestrated changes in transcription and are determined by transcription factors (TFs) binding to accessible DNA regulatory elements found in promoters and enhancers. Ubiquitously expressed TFs such as nuclear factor-κB (NF-κB), interferon regulatory factors (IRFs), and activator protein 1 (AP.1) each play a central role in eliciting an inflammatory response to extracellular Toll-like receptor 4 (TLR4) stimulation by lipopolysaccharide (LPS). These ubiquitous stimulus-inducible TFs appear to work in conjunction with lineage-restricted constitutive TFs, such as PU.1, to define lineage-specific enhancers (Ghisletti et al., 2010; Heinz et al., 2010). However, the regulatory logic underlying the activation of specialized gene expression programs is to a large extent unknown. TFs that respond to tissue-specific microenvironmental cues and fine-tune cellular identities (Ostuni and Natoli, 2011) add to the complexity of this regulatory logic.

In this context, we demonstrated that IRF5 is critical in establishing inflammatory phenotypes in vitro and is involved in the positive regulation of type 1 T helper (Th1)/Th17-associated mediators, such as interleukin-1 (IL-1), IL-12, IL-23, and tumor necrosis factor α (TNF-α) (Krausgruber et al., 2010, 2011). Moreover, IRF5 is capable of repressing anti-inflammatory genes associated with the macrophage colony-stimulating factor (M-CSF)-derived phenotype, such as *IL-10* (Krausgruber et al., 2011). A functional consequence of this dual role is demonstrated by studies showing that IRF5 is essential in the development of Th1 responses to *Leishmania donovani* infection (Paun et al., 2011) and in the susceptibility to lethal endotoxic shock (Takaoka et al., 2005). These divergent functions of IRF5 suggest that IRF5 cooperates with different cofactors at inflammatory versus homeostatic gene regulatory elements. In fact, we have reported that IRF5 forms a protein complex with NF-κB RelA to drive a sustained induction of the human *TNF* gene (Krausgruber et al., 2010).

In this study, we used GM-CSF (granulocyte/macrophage-colony-stimulating factor)-derived macrophages (GM-bone marrow-derived macrophages [BMDMs]) to investigate whether the recruitment of IRF5 via its interactions with RelA is a common mechanism of proinflammatory gene regulation by IRF5. By intersecting the chromatin immunoprecipitation-sequencing (ChIP-seq) analysis of the individual TFs in LPS-stimulated GM-BMDMs with gene expression data and histone methylation status data sets, we show that the IRF5 and RelA cistromes target inflammatory genes. The two cistromes overlap only at a limited number of genomic regions located in the PU.1-marked regulatory elements of inflammatory genes, 70% of which are induced upon LPS stimulation, as shown by the recruitment of RNA polymerase II (PolII). Using in vivo and in vitro motif discovery analyses, we demonstrate that the IRF5:RelA cistrome is best explained by the presence of consensus NF-κB and noncanonical composite PU.1:interferon-stimulated response element (ISRE)-binding sites. We demonstrate that IRF5 genome recruitment to inflammatory genes is aided by RelA. These results reveal a genomic strategy for controlling an inflammatory gene program in GM-BMDMs via establishment of a unique IRF5:RelA cistrome to target inflammatory genes.

## Results

### Genome-wide Alignment of IRF5 and RelA Binding in GM-CSF BMDMs

To investigate the model of IRF5-RelA transcriptional cooperation, ChIP-seq was used to determine the genome-wide binding of IRF5, RelA, and PolII in GM-BMDMs stimulated with LPS or left unstimulated. Upon LPS stimulations, these macrophages are predominantly homogeneous IRF5-positive cells that display a distinct phenotype of cytokine and cell surface molecule expression compared to M-CSF (CSF-1) (M)-BMDMs (Figure S1A) (Fleetwood et al., 2007; Weiss et al., 2013). ChIP-seq libraries were prepared for untreated cells or cells treated for 0.5 or 2 hr with LPS. Nonimmunoprecipitated input DNA isolated under the same conditions was also subjected to sequencing. Enriched bound genomic regions (peaks) were identified using the ZINBA (zero-inflated negative binomial algorithm; Rashid et al., 2011) at a false discovery rate (FDR) of 1% (Table S1A). We identified 1,252, 6,052, and 8,805 RelA peaks (RelA cistrome) and 3,591, 4,157, and 4,213 IRF5 peaks (IRF5 cistrome) at 0, 0.5, and 2 hr, respectively, post-LPS stimulation (Table S1B). The scatterplot analysis of the data sets demonstrated a strong influence of LPS stimulation on both RelA and IRF5 recruitment (Figures S1B and S1C). We also found an 80% overlap of RelA peaks identified in this study to peaks in GM-CSF-derived dendritic cells (GM-bone marrow dendritic cells [BMDCs]) (Garber et al., 2012). As a control for the IRF5 data set, we performed IRF5 ChIP quantitative PCR (ChIP-qPCR) in IRF5 knockout (KO) and wild-type (WT) cells stimulated with LPS and demonstrated a specific enrichment in IRF5 binding at the ChIP-seq-identified peaks in WT cells (Figure S1C). We also identified 1,679 and 4,879 PolII peaks 0.5 and 2 hr, respectively, post-LPS stimulation.

**Figure 1:**
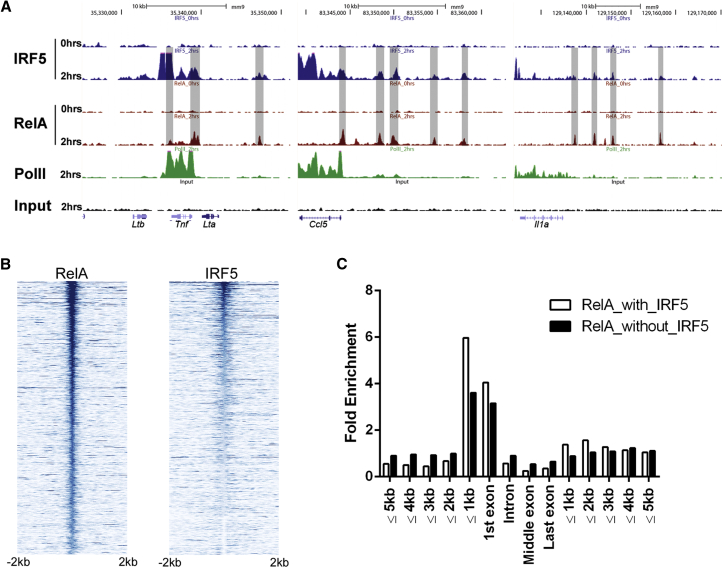
Distribution of IRF5- and RelA-Bound Regions in the Genome following LPS Stimulation (A) Representative USCS Genome Browser tracks in the *Tnf*, *Ccl5*, and *Il-1a* loci for IRF5 (blue), RelA (red), PolII (green), and Input (black) of unstimulated (0 hr) or LPS-stimulated (2 hr) GM-BMDMs. Overlapping IRF5 and RelA regions are highlighted in gray. (B) IRF5 reads colocalize with RelA peaks. The ChIP-seq data sets for RelA and IRF5 (±2 kb) were each aligned with respect to the center of the RelA peaks and sorted by the height of the RelA-marked regions. Each line represents a RelA peak. A total of 1,000 representative peaks are shown. (C) Fold enrichment of RelA ChIP-seq peaks with (white bars) or without (black bars) IRF5 was aligned to the nearest gene structures in which 5 kb upstream/downstream regions were split into 1 kb windows. A 5.2-fold enrichment of overlapping RelA and IRF5 peaks at the proximal 1 kb region to 3.7-fold for RelA peaks that do not co-occur with IRF5 (p = 10^−4^, as defined by simulation procedure; see Experimental Procedures). See also Figure S1 and Table S1.

Some illustrative binding regions are shown in Figure 1A, including the lymphotoxin-α (*Lta*), *Ltb*, and *Tnf* gene cluster, chemokine (C-C motif) ligand 5 (*Ccl5*), IL-1α (*Il-1a*), and other immune related gene loci. In the case of the *Tnf* gene cluster, IRF5 and RelA show similar binding patterns as we previously reported in human monocyte-derived dendritic cells (Krausgruber et al., 2010). The peaks called by the ZINBA in these representative regions indicate considerable overlap of IRF5 and RelA binding to this gene locus. In general, IRF5 binding was found to co-occur frequently with RelA binding (801 peaks at 1% FDR) (Figure 1B). Using a simulation procedure that controls for genomic background (Ponjavic et al., 2009), we observe that the overlap between RelA- and IRF5-binding sites is 3.4-fold greater than expected (p < 10^−4^). Therefore, the overlap of IRF5 and RelA binding initially observed in the *Tnf* gene cluster (Krausgruber et al., 2010) (Figure 1A) reflects a genome-wide phenomenon. Of interest, a significant enrichment in co-occurrence of RelA peaks with another member of the IRF family, IRF1, but not with IRF2 or IRF4, was observed in a high-throughput analysis of TF binding in GM-BMDCs (Garber et al., 2012).

Using the simulation procedure described above, we observed a 21-fold (p < 10^−4^) increase in IRF5 recruitment following LPS stimulation for 2 hr (Table S1C). IRF5 peaks were also observed downstream of the 3′ UTRs of protein-coding genes (Table S1C; Figure S1D), with 33% of IRF5-targeted genes containing an IRF5 peak downstream of the transcription end site (Figure S1E). Binding of RelA upstream of transcription start sites (TSSs) amounted to 19% of all peaks at 2 hr post-LPS stimulation, but no significant enrichment in binding to intergenic, intronic, and downstream regions was observed (Table S1D). Correspondingly, the proportion of RelA peaks that co-occur with IRF5 peaks (IRF5:RelA cistrome) is markedly increased in the proximal 1 kb regions upstream of the TSS (5.2-fold change; p < 0.0001; Figure 1C). In summary, these analyses highlight that up to 20% of IRF5- and RelA-binding events occur within a limited part of the genome, namely the relatively short regions just upstream of protein-coding genes.

### IRF5 and RelA Cistromes Intersect at PU.1-Marked Regulatory Elements of LPS-Induced Genes

**Figure 2:**
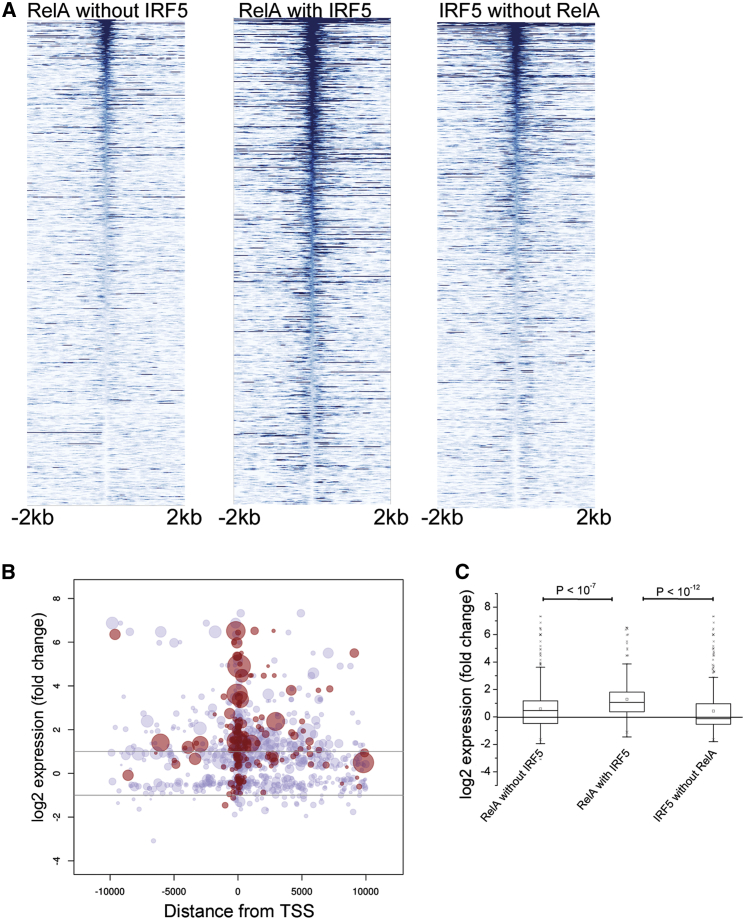
IRF5 and RelA Colocalize at TSSs of Positively Regulated Genes (A) IRF5 and RelA complex colocalizes with PolII peaks. The ChIP-seq reads for RelA without IRF5, RelA with IRF5, and IRF5 without RelA were each aligned with respect to the center of the PolII peaks and sorted by the height of the PolII-marked regions. A total of 500 representative peaks are shown. (B) Bubble plot representation of RelA-binding sites around differentially regulated genes. The plots indicate the position of ChIP-seq peaks with respect to the closest TSS (x axis) and the observed fold change (y axis) in microarray expression experiments. The size of a bubble denotes the strength of the ChIP-seq peak. Red bubbles indicate RelA with IRF5; blue bubbles indicate RelA without IRF5. (C) Box plot of gene expression fold change differences upon LPS stimulation with three categories of binding events in their vicinity as indicated. The fold change differences are significant (Mann-Whitney U test). See also Figure S2 and Table S2.

To understand whether the binding of IRF5 and RelA influences PolII recruitment, we analyzed the degree of genome-wide overlap between IRF5 and RelA with PolII occupancy following 2 hr of LPS stimulation. PolII peaks overlapped with the IRF5:RelA cistrome (>116-fold over genomic background; p < 10^−4^) more prominently than with the rest of either RelA or IRF5 peaks (Figure 2A). Analysis of the degree of PolII overlap with TSSs demonstrated increased overlap with TSSs when both IRF5 and RelA bind the gene upstream region (Figure S2A). Moreover, when we combined IRF5- and RelA-binding data with microarray gene expression data at the same time point, we noted that the peaks of the IRF5:RelA cistrome were centered around the TSS of strongly upregulated genes, whereas the RelA peaks that did not overlap with IRF5 displayed more uniform distribution in both upregulated and downregulated genes around the TSS (Figure 2B). Further analysis of gene expression across the stratified ChIP-seq peaks revealed that genes targeted by both RelA and IRF5 were significantly more upregulated than either RelA (p < 10^−7^) or IRF5 (p < 10^−12^) acting independently (Figure 2C). Among 340 strongly (>2-fold; FDR, 1%) upregulated genes, 74 were targeted by both RelA and IRF5 (Table S2C). RelA binding explains a similar number of upregulated genes, whereas IRF5 explains fewer. Of interest, the individual presence of IRF5 at the gene promoter explains a much larger proportion of 202 strongly downregulated (>2-fold; FDR, 1%) genes (Table S2A).

Next, we examined whether the RelA and IRF5 cistromes show characteristic chromatin signatures of functional genomic elements, i.e., enhancers and promoters marked by relatively high levels of monomethylation or trimethylation of lysine 4 of histone 3 (H3K4), respectively. To do this, we intersected the data sets with H3K4me1/H3K4me3-positive regions from M-BMDMs (Heinz et al., 2010) and GM-BMDCs (Garber et al., 2012). IRF5 and RelA peaks associated with both H3K4Me1 and H3K4Me3 chromatin marks (p < 10^−4^; Table S2B).

It has recently been shown that binding of a pioneer TF PU.1 is essential for defining macrophage-specific enhancers because it promotes the deposition of H3K4me1 (Ghisletti et al., 2010; Heinz et al., 2010). Here, we examined the aggregated densities of PU.1 reads reported in Garber et al. (2012) over IRF5:RelA peaks and found that they align perfectly with the peaks of H3K4me1 and H3K4me3 deposition at enhancers and promoters, respectively, with the notable bimodal distribution of histone marks indicative of nucleosome depletion (Figure S2A). Moreover, binding of IRF5:RelA peaks occurred at both PU.1-marked promoters and enhancers (p < 10^−4^; Table S2C). Thus, the genes that expression is strongly induced in macrophages by LPS are under the control of the IRF5:RelA cistrome, which is centered around the TSS.

### The IRF5:RelA Cistrome Targets Regulatory Elements of Key Inflammatory Genes

**Figure 3:**
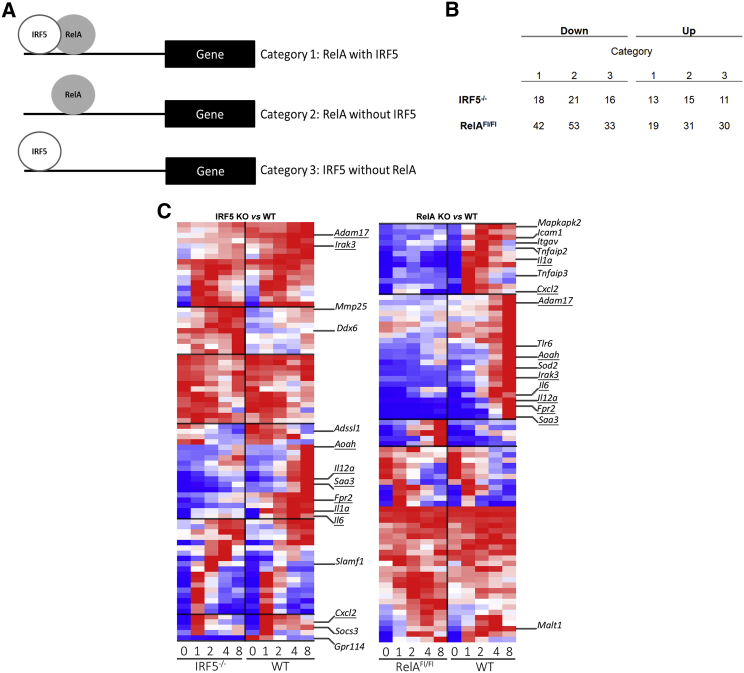
Association of IRF5:RelA-Bound Genes with Immune Genes (A) Schematic of promoters bound by IRF5 and RelA. Each promoter (10 kb upstream and 0.5 kb of the TSS) containing IRF5 and/or RelA ChIP-seq peaks was classified into (1) bound by both IRF5 and RelA, (2) bound by RelA but not IRF5, or (3) bound by IRF5 but not RelA. (B) Genome-wide profiling of LPS-affected genes in GM-BMDMs from either conventional IRF5 KO or conditional RelA KO (RelA Fl/Fl Mx1Cre; in which Mx1-Cre expression was induced by PolyI:C) compared to WT. Number of genes in each category significantly affected by KO of IRF5 or RelA (>2-fold; FDR, 1%) is shown (Down, regulator induces gene; Up, regulator represses gene). IRF5 KO resulted in 164 downregulated genes and 99 upregulated genes compared to WT. RelA KO resulted in 263 downregulated genes and 236 upregulated genes compared to WT. (C) Gene expression heatmaps of category 1 genes. GM-BMDMs from conventional IRF5 KO (left panel) or conditional RelA KO each compared to WT controls following stimulation by LPS for 0, 1, 2, 4, or 8 hr are shown; underlining indicates genes that are affected in both IRF5 and RelA KO. (Data are pooled from three experiments; blue to red represents increase level of gene expression.) See also Figure S3 and Table S3.

To identify genes that are directly and functionally affected by either the IRF5:RelA cistrome or IRF5 and RelA acting individually, we first categorized promoters (up to 10 kb upstream and 0.5 kb of the TSS) of the genes into three categories. These consist of genes that encompass ChIP-seq peaks present in (1) both IRF5 and RelA, (2) only RelA, or (3) only IRF5 (Figure 3A). We next performed global expression profiling to identify LPS-affected genes that are differentially expressed in either GM-BMDMs from IRF5 conventional KO or conditional RelA KO (RelA Fl/Fl Mx1Cre) (Luedde et al., 2008) compared to WT mice (Figure 3B). The expression profiles of GM-BMDMs at 0, 1, 2, 4, and 8 hr after LPS stimulation were analyzed for differential gene expression. IRF5 or RelA deficiency resulted in inhibition of a very selective subset of category 1 genes that encompassed key inflammatory mediators (defined as Panther Pathway “Inflammation mediated by chemokine and cytokine signaling pathway”; Hyper FDR, q <10^−10^), such as *Il-6*, *Il-12a*, *Il-1a*, *Fpr2*, *Aoah*, *Adam17*, *Cxcl2*, and *Saa3* (Figure 3C). Moreover, expression of some other important inflammatory genes was affected by either only IRF5 deletion (*Mmp25* and *Socs3*) or only RelA (e.g., *Tnfaip3*, *Itgav*, *Malt1*, *Icam1*, and *Sod2*) (Figure 3C). In category 2 genes, we observe that RelA, but not IRF5, KO has a direct effect on expression at, for example, *Lcn2*, *Fas*, *H2-M2*, *Clec4a1*, *Casp7*, and *Nlrp3* gene loci (Figure S3; Table S3). In category 3 genes, we observe that the lack of IRF5, but not RelA, has affected expression of, for example, *Nos2*, a key marker of M1 activity (Figure S3; Table S3). The observation that the expression of some genes in category 2 is affected by IRF5 KO and some genes in category 3 by RelA KO indicates an indirect effect via a secondary regulator in a feedforward loop (Mangan and Alon, 2003). In total, we find that 263 and 499 transcriptionally active genes are affected by the lack of IRF5 or RelA, respectively. Consistent with the published data demonstrating that most RelA target sites were not associated with transcriptional changes (Lim et al., 2007), at many loci, IRF5 or RelA binding did not directly correlate with change in gene expression, even in the absence of a TF. It is possible that previously noted “billboard” organization of immune genes’ promoters (Garber et al., 2012) allows for a degree of redundant recruitment of TFs. Hence, the combined global profiling of IRF5- and RelA-bound sites and gene expression in cells deficient in either TF has highlighted the direct role of the IRF5:RelA cistrome in transcriptional regulation of selective key inflammatory genes.

### The Presence of Canonical κB and Composite PU.1:ISRE Sites Is Characteristic of the IRF5:RelA Cistrome

**Figure 4:**
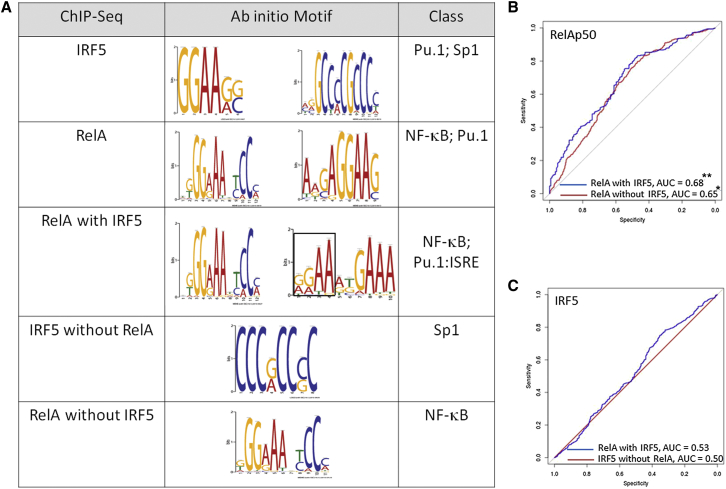
Enrichment of PBM-Determined κB Sites, but Not ISREs, in ChIP-Seq Peaks (A) A parallel version of Multiple EM for Motif Elicitation-ChIP (Machanick and Bailey, 2011) was used to perform a de novo sequence search in the five subtypes of ChIP binding using 500 top-binding sequences in each category. The PU.1 motif (GGAA) and Sp1 motif were derived for the entire IRF5 data set. The κB and PU.1 motifs were derived for the entire RelA data set. The κB and composite PU.1 (boxed):ISRE motifs were derived for overlapping RelA and IRF5 peaks. The Sp1 motif was derived for the IRF5 data set in the absence of RelA, and the κB site was derived for the RelA data set in the absence of IRF5. (All motifs shown have an e value of <10^−5^.) (B) ROC curve analysis quantifying enrichment within RelA-bound regions (blue indicates with IRF5; red indicates without IRF5) of RelA-p50 PBM-determined sites. (C) ROC curve analysis quantifying enrichment within IRF5-bound regions (blue indicates with RELA; red indicates without RelA) of IRF5 PBM-determined ISREs. AUC values quantify enrichment. (Wilcoxon-Mann-Whitney U test, ^∗∗^p < 10^−29^; ^∗^p < 10^−7^.) See also Figure S4.

To better understand the regulatory code at inflammatory loci, we first performed ab initio DNA motif analysis around the top 500 RelA and IRF5 peaks. Motif analysis revealed the known κB motif (Natoli et al., 2011; Siggers et al., 2012) in RelA peaks (Figure 4A). The same analysis of IRF5 peaks found no motifs similar to known canonical ISRE, A/GNGAAANNGAAACT (Badis et al., 2009; Tamura et al., 2008) (Figure 4A). However, the PU.1-binding motif was the top-scoring motif enriched in the IRF5 peaks (Figure 4A). The other top-binding motif in this data set included TFs recognizing CpG-rich sequences that are associated with gene promoters, such as Sp1 (Figure 4A). Interestingly, the binding regions co-occupied by IRF5 and RelA were enriched in κB site and binding motif that resembled a composite PU.1:ISRE, with the PU.1 site (Figure 4A, boxed) adjacent to the ISRE half-site (Figure 4A). This site was previously reported for immune cell development-related IRF4 and IRF8 (Brass et al., 1996; Escalante et al., 2002; Tamura et al., 2005). Additionally, whereas RelA binds to the κB site in the absence of IRF5, the latter is likely to bind to the CpG-rich sequence SP1 in the absence of RelA (Figure 4A). Thus, the mode of IRF5 in vivo binding groups it with immune cell development-related IRFs and strongly suggests that such IRF proteins exert their function as cofactors and not individually.

Because ChIP only identifies genomic regions that interact with TFs but not necessarily individual binding sites (Gordân et al., 2009; Jolma et al., 2010; Wong et al., 2011), we used protein-binding microarrays (PBMs) for purified recombinant IRF3 and IRF5 protein to map the site of TF-DNA interactions with precision. For comparison, PBM data for RelAp50 were used from Wong et al. (2011). We analyzed 3,072 12-mer sequences designed around the ISRE consensus (see Experimental Procedures) carrying 4 different flanks. The sequences were ranked and used to produce binding motifs. The logo emerging from the top 50 binders was very similar to the one obtained by Badis et al. (2009), whereas the top 500 sequences produced a motif that was less stringent in positions 6, 9, and 11 (Figure S4, bottom panel).

All the IRF5- and RelAp50-binding sequences with their respective *Z* scores were used to perform receiver operating characteristic (ROC) curve analyses using the IRF5 and RelA cistromes from this study to quantify whether the IRF5- or RelA-bound regions (true positives) scored higher than the unbound regions (true negatives), similar to Siggers et al. (2012). We observed equally large areas under the ROC curve (AUC) when RelAp50 kmers were used to explain the IRF5:RelA cistrome peaks (AUC, 0.68) or the rest of RelA peaks (AUC, 0.65) compared to true negatives (Figure 4B). Thus, we concluded that RelAp50 binds to sequences in vivo that resemble its DNA-binding preferences in vitro. In contrast, AUC enrichment scores for IRF5 kmers demonstrated low nondiscriminating enrichment scores for the peaks of the IRF5:RelA cistrome (AUC, 0.53) and the rest of IRF5 peaks (AUC, 0.50) (Figure 4C).

Together with the ab initio analysis above, we therefore interpret this result as evidence that at the inflammatory gene loci, IRF5 is likely to be recruited to a composite PU.1:ISRE site rather than to a canonical ISRE site, whereas RelA binds directly to the respective κB site. The dynamics of PU.1 and IRF5 binding at the PU.1:ISRE composite sites are unclear and warrant further investigation.

### RelA Aids in IRF5 Recruitment to Promoters of Inflammatory Genes

**Figure 5:**
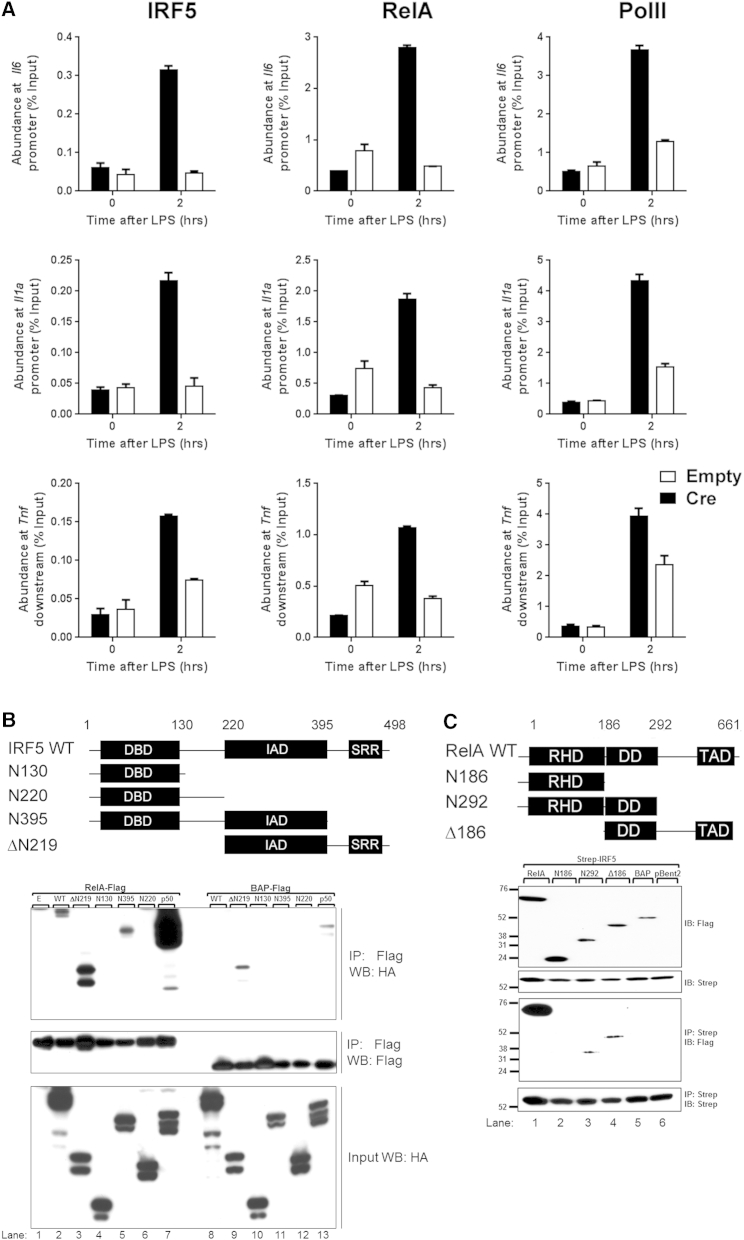
IRF5:RelA Interaction Occurs via the IRF IAD and RelA DD (A) GM-BMDMs from RelAFl/Fl mice were infected with either Cre or Empty adenovirus and stimulated with LPS (100 ng/ml) for 2 hr or left unstimulated and used in ChIP analysis for IRF5, RELA, or PolII recruitment on *Il-6*, *Il-1a*, and *TNF* loci. Data indicate mean percent input relative to genomic DNA ± SD of a representative experiment. (B) Schematic of IRF5 truncation mutants prepared by PCR using IRF5 cDNA as a template. Each mutant was cloned into pBent vector with One-STrEP and HA tags at the N terminus. HEK293-TLR4-Md2/CD14 cells were cotransfected with either RelA-Flag (lanes 1–7) or Bap-Flag (lanes 8–13) and each of the IRF5 truncation mutants (lanes 2–6 and 8–12) or p50 (lane 7 and 13) as a positive control. Following immunoprecipitation (IP) on M2 anti-Flag beads, the Flag peptide eluates were immunoblotted for IRF5 truncation mutants (anti-HA antibody; top panel) and bait (anti-Flag antibody; middle panel). Immunoblots of input lysate show equal expression of bait IRF5 truncation mutants (anti-HA; bottom panel). RelA interacts with IRF5 WT (lane 2) and truncation mutants possessing the IAD (lanes 3 and 5), but not mutants lacking the IAD (lanes 4 and 6). WB, western blot. (C) Schematic of RelA truncation mutants prepared by PCR using IRF5 cDNA as a template. Each mutant was cloned into pBent vector with FLAG tag at the N terminus. HEK293-TLR4-Md2/CD14 cells were cotransfected with full-length RelA-FLAG (lane 1), truncated RelA-FLAG mutants (lanes 2–3), BAP-FLAG (lane 4), or pBent (lane 5) and One-STrEP-IRF5. Immunoblots of input lysate show equal expression of prey RelA truncation mutants (anti-FLAG; bottom panel). Following affinity purification on Strep-Tactin beads, the biotin eluates were immunoblotted for RelA truncation mutants (anti-FLAG; top panel). Immunoblots of the eluates show equal recovery of the bait One-STrEP-IRF5 (middle panel). IRF5 interacts with RelA WT and truncation mutants containing DD (lanes 1, 3, and 4), but not mutants lacking the DD (lane 2). See also Figure S5.

We previously reported that IRF5 can functionally interact with RelA at the human TNF locus (Krausgruber et al., 2010). Here, we addressed the question whether recruitment of IRF5 to inflammatory gene loci is commonly mediated by RelA. We examined IRF5 recruitment to the selected genes, expression of which was shown to be directly dependent on IRF5 (Figures 3B and S5A) in cells with depleted levels of RelA. GM-BMDMs were generated from the bone marrow of RelAFl/Fl mice (Luedde et al., 2008) and infected with Cre-expressing adenovirus. Efficiency of RelA deletion was about 50% as judged by analysis of residual RelA protein by western blot (Figure S5B). Following stimulation with LPS for 2 hr, recruitment of IRF5, as well as RelA and PolII, to the *Il-1a*, *Il-6*, and *Tnf* genomic loci was analyzed by ChIP-qPCR. We observed a significant reduction in IRF5 binding to the regions of overlapping IRF5:RelA peaks (Figure 5A). Thus, together with the previously observed dependence of IRF5 binding to the 3′ region of the human TNF gene (Krausgruber et al., 2010), our results suggest that recruitment of IRF5 to DNA at inflammatory gene loci is assisted by RelA.

To map the interacting domains, we generated in-frame One-STrEP and HA-tagged truncation mutants of the key domains of IRF5 (Figure 5B, top panel) and FLAG-tagged truncation mutants of the key domains of RelA (Figure 5C, top panel). IRF5 truncation mutants or p50 (as positive control) was coexpressed to equal levels in HEK293-TLR4-CD14/Md2 cells along with RelA-Flag or BAP-Flag as negative control. The resulting lysates were subjected to One-STrEP immunoprecipitation, and the precipitated proteins were analyzed by immunoblotting with anti-HA antibody. The truncation mutants IRF5-ΔN219 and IRF5-N395 were comparable to the WT protein in binding RelA-Flag. In contrast, removal of the IRF association domain (IAD) in truncation mutants IRF5-N130 and IRF5-N220 resulted in impaired binding to RelA-Flag (Figure 5B, bottom panel). Flag-tagged RelA truncation mutants or BAP-Flag as negative control was coexpressed to similar levels along with One-STrEP-IRF5, subjected to One-STrEP immunoprecipitation, and the precipitated proteins were analyzed by immunoblotting with anti-FLAG antibody. The removal of the dimerization domain (DD) in truncation mutant RelA-N186 resulted in impaired binding to One-STrEP-IRF5 (Figure 5C, bottom panel). Thus, IRF5 IAD and RelA DDs are critical for IRF5-RelA interactions.

## Discussion

An emerging view on the transcriptional networks that dictate the response of macrophages while encountering microbial stimuli is that they consist of pioneer lineage-specific (e.g., CEBPβ, PU.1), basal (e.g., JunB, ATF3), and stimulus-inducible (NF-κB, IRFs, AP.1) TFs (Garber et al., 2012). Here, we investigated how TFs that define functional macrophage specialization (Garber et al., 2012; Ghisletti et al., 2010; Heinz et al., 2010), such as IRF5, contribute to the determination or regulation of specific subsets of the regulatory elements. We demonstrate that IRF5 is recruited to such elements of LPS-induced inflammatory genes and is essential for their efficient transcription. We find that NF-κB RelA assists IRF5 in binding to DNA, and the two factors set up a unique “inflammatory” IRF5:RelA cistrome. We also map the interface of IRF5:RelA interactions, paving the way to possible new therapeutics, that would specifically reduce inflammatory activities, without having deleterious effects on the whole innate immunity that is an essential first line of defense against microbes.

Binding of a pioneer TF, PU.1, in macrophages is thought to be sufficient to promote the deposition of H3K4me1 and to create small open regions of accessible DNA that can be bound by stress-inducible dynamic TFs, such as NF-κB and IRFs (i.e., enhancers) (Natoli et al., 2011). Less clear is whether PU.1 also promotes the deposition of H3K4me3 and chromatin opening at gene promoters. We find that PU.1 binding corresponds to the peaks of both H3K4me1 and H3K4me3 deposition and demonstrates bimodal distribution of histone marks indicative of nucleosome depletion. Binding of IRF5:RelA is significantly enriched on PU.1-marked regulatory elements. This is in line with previous studies that indicated that a subset of macrophage PU.1-marked enhancers was enriched for both NF-κB and IRFs (Ghisletti et al., 2010). Moreover, we find that the IRF5:RelA cistrome encompasses a noncanonical composite PU.1:ISRE. We observed that IRF5 can also physically interact with PU.1 (data not shown), whereas others recently demonstrated that a preferred mode of IRF5 binding may actually be to a half ISRE site (Jolma et al., 2013). More work is needed to tease out the dynamics of RelA and PU.1 involvement in the binding of IRF5, but it is highly likely that PU.1 binds first because it functions as a pioneering factor in macrophages (Garber et al., 2012; Ghisletti et al., 2010; Heinz et al., 2010), followed by the binding of RelA at inflammatory gene loci and subsequently docking of IRF5. Thus, in inflammatory macrophages, IRF5 imposes yet another previously unobserved level of control on the transcriptional network.

Can this be a mode of binding at other gene loci identified in this study as IRF5 but not RelA targets? The interactome of IRF5 is rapidly expanding (Eames et al., 2012; Feng et al., 2010); thus, it is possible that other yet to be identified TFs may aid to recruit IRF5 to other gene promoters in the absence of RelA binding. We found a strong enrichment in SP1-binding motif under IRF5 ChIP-seq peaks, suggesting that this factor might be involved in high-order transcriptional complexes containing IRF5. Supporting this model is a recent analysis of IRF5 binding in human peripheral blood mononuclear cells stimulated with immune complexes that also identified Sp1 as one of the major motifs in IRF5 target regions (Wang et al., 2013).

It is possible that IRF5 acts similarly to another member of the IRF family, IRF3, which was shown to promote transcription by removing a nucleosome barrier (Ramirez-Carrozzi et al., 2009). IRF5 interacts with a number of chromatin modifiers including acetyltransferases (CBP/300), which were specifically recruited to the interferon α promoter in response to viral induction (Feng et al., 2010). However, it is unlikely that IRF5 plays a causative role in the initial chromatin remodeling because binding of NF-κB, which assists IRF5 in recruitment to inflammatory gene loci, requires nucleosome-free DNA (Lone et al., 2013; Natoli, 2012). Can IRF5 play a role in chromatin remodeling at later stages of gene expression? We have recently demonstrated that IRF5 interacts with KAP1 to indirectly recruit SETDB1 methyltransferase, ultimately leading to deposition of H3K9me3—a mark of transcriptional repression (Eames et al., 2012).

IRF5 is a genetic risk factor for many autoimmune diseases, including systemic lupus erythematosus, rheumatoid arthritis, multiple sclerosis, and inflammatory bowel disease (Dideberg et al., 2007; Dieguez-Gonzalez et al., 2008; Graham et al., 2006; Kristjansdottir et al., 2008). Anti-inflammatory drugs that target molecules that are pivotal to the inflammatory process, like TNF and COX2, have proved successful, but the ultimate aim would be to target transcription of a specific subset of proinflammatory genes (Smale, 2010). Inhibiting IRF5 activity may pave the way for the development of more selective drugs targeting the basic mechanisms underlying the inflammatory response.

## Experimental Procedures

### Mice

The generation of *Irf5*^−/−^ mice has been described by Takaoka et al. (2005). All procedures were approved by the Ethical Review Process Committee and the UK Home Office, in accordance with the Animals (Scientific Procedures) Act 1986.

### Cell Culture

For the generation of M1 macrophages differentiated with GM-CSF, bone marrow of WT C57Bl6 mice was cultured in RPMI-1640 medium (PAA Laboratories) supplemented with recombinant mouse GM-CSF (20 ng/ml; PreproTech). After 8 days, cells were washed with PBS and replated, then stimulated with LPS (100 ng/ml; Alexis Biochemicals).

### ChIP-Seq

Nuclear lysates of formaldehyde-fixed GM-CSF macrophages were isolated as described previously by De Santa et al. (2007). Each lysate was immunoprecipitated with 10 μg of the following antibodies: IRF5 (Abcam; ab21689), RelA (Santa Cruz Biotechnology; sc-372), and PolII (Santa Cruz; sc-899). ChIP was performed for each antibody as described previously by Ghisletti et al. (2010).

Please note that independent IRF5 ChIP-seq data sets were recently generated by us in WT and IRF5^−/−^ GM-BMDMs with 50 bp paired-end sequencing following stimulation with LPS for 0 and 2 hr. Preliminary analysis of these data sets by MACS2 algorithm with filtering out of peaks detected in the IRF5^−/−^ from the WT corroborated the findings reported in this manuscript. Further details of next-generation sequencing and analysis are provided in the Supplemental Experimental Procedures.

### Microarray Analysis

Microarray (accession number E-MTAB-2032) data were analyzed in R/bioconductor using the beadarray (version 2.4.2; Dunning et al., 2007). Differentially expressed genes were called with SAM method (Tusher et al., 2001) applying an FDR threshold of 10%.

## Author Contributions

D.G.S. designed research, performed the wet lab experiments, and wrote the paper. A.H. performed bioinformatics analysis of the data and helped write and edit the manuscript.

## Accession Numbers

The ArrayExpress accession numbers for the microarray data, single-end ChIP seq data, and paired-end ChIP-seq data reported in this paper are, respectively, E-MTAB-2032, E-MTAB-2031, and E-MTAB-2661.

## Supplemental Information

Document S1. Supplemental Experimental Procedures, Figures S1–S5, and Tables S1–S3

Document S2. Article plus Supplemental Information
