# Supplementary material for: IRF5:RelA Interaction Targets Inflammatory Genes in Macrophages
Source: Cell Rep. 2014 Aug 21;8(5):1308–17. doi: 10.1016/j.celrep.2014.07.034 (PMC4471814; doi:10.1016/j.celrep.2014.07.034)
Supplement: Document S1. Supplemental Experimental Procedures, Figures S1–S5, and Tables S1–S3 [file mmc1.pdf]

Supplemental Experimental Procedures

Plasmids

Expression construct encoding full-length IRF5 with HA and Onestrep-tags were described in Krausgruber et al (2010). The plasmids encoding IRF5 N220, IRF5ΔDBD and IRF5ΔN219 are described in Eames et al., 2012. HA-tagged deletion mutants - IRF5 N130 (aa 1-130) and N395 (aa 1-395) - were amplified by PCR from the full-length human IRF5 cDNA and inserted into pBent2-strep vector. Flag-tagged deletion mutants – RelA N186 (aa1-186), RelA N292 (aa1-292) and RelA Δ186 (aa186-551) – were amplified by PCR from plasmid DNA encoding full-length RelA-Flag (described in Krausgruber et al., 2010), and inserted into the pBent2 vector. All constructs were verified by DNA sequencing. The sequences and restriction maps are available upon request.

Cell Culture

For the generation of M1 macrophages differentiated with GM-CSF, bone marrow of wild type C57Bl6 mice was cultured in RPMI-1640 medium (PAA Laboratories) supplemented with recombinant mouse GM-CSF (20 ng/ml; Preprotech). After 8 d, cells were washed with PBS and replated, then stimulated with LPS (100 ng/ml; Alexis Biochemicals).

Adenoviral Cre-mediated Rela knockdown

For the Cre-mediated conditional knockdown of RelA, M1 macrophages from RelAFl/Fl mice were differentiated as described above. After 7 days cells were washed with PBS and replated in 10 cm plates (10^7 cells per plate) in antibiotics-free RPMI containing either Cre or empty vector (pBent2) adenoviral particles at a multiplicity of infection (MOI) of 50:1 in a final volume of 5 mL. The plates were incubated for 4hrs at 37°C followed by the addition of 5mL standard media per plate. Cells were allowed to recover for a further 12 hrs before being left unstimulated or stimulated with LPS (100 ng/ml; Alexis Biochemicals).

ChIP-seq

50, 100 and 300 million GM-CSF derived M1 macrophages were used for each Pol II, RelA and IRF5 ChIP experiment, respectively. Cells were fixed for 10

minutes with 1 % formaldehyde, quenched with 125 mM of Tris pH 7.5 and washed with ice-cold PBS. Nuclear lysates were isolated as described previously (De Santa et al. 2007) and sonicated with a Bioruptor (Diagenode) to obtain chromatin fragment sizes that average 500bp. Each lysate was immunoprecipitated with 10 µg of the following antibodies: IRF5 (Abcam; ab21689), RelA (Santa Cruz; sc-372) and PolII (Santa Cruz; sc-899). ChIP was performed for each antibody as described previously (Ghisletti et al. 2010). ChIPped DNA was quantified with the Quant-iT dsDNA High Sensitivity Assay Kit (Invitrogen #Q33120). DNA yields ranged from 10 – 20 ng. The ChIP-Seq datasets were generated using 33bp single end sequencing (Accession number: E-MTAB-2033). **NOTE:** independent IRF5 ChIP-seq datasets were recently generated by us in WT and IRF5-/- GM-BMDMs with 50bp paired end sequencing following stimulation with LPS for 0 and 2hrs in duplicate. 13-24 (mean = 22) million reads were mapped to the genome for each experimental condition. (Accession number: E-MTAB-2661). Using MACS2 algorithm at 20% FDR we detected 417 and 533 peaks in duplicates of IRF5+/+ datasets, and 461 and 457 peaks in duplicates of IRF5-/- datasets at 0h. Following 2h of LPS stimulation 1453 and 2345 peaks were detected in duplicates of IRF5+/+ datasets, while the number of peaks in IRF5-/- datasets remained largely unchanged (319 and 372 peaks). Combining IRF5+/+ datasets at 2 h post stimulation amounted to 2835 peaks, while combining IRF5-/- dataset resulted in 497 peaks. Thus, ~15% of IRF5 ChIP-Seq peaks were false positive, with remaining 2538 IRF5 binding peaks being bona fide peaks. Preliminary cross-validation analyses using this dataset corroborated the findings reported in this manuscript.

ChIP-qPCR of Cre-mediated RelA Knockdown

A total of $1x10^7$ Cre or Empty adenovirus infected cells described above were used for ChIP-qPCR. Sonicated nuclear lysates were prepared as in ChIP-seq procedure described above. Each lysate was immunoprecipitated with 3 µg of either Pol II, or IRF5 or RelA. The immunoprecipitated DNA fragments were then interrogated by real-time PCR using SYBR Premix Ex Taq II master mix (Takara Bio) and the following primers: Il1a (TGCCCAGTCTGTCCCTCCTCATGCT and CCCGAGCTTTGGCTCCAGTCTGCT); Il1b (GGATGTGCGGAACAAAGGTAGGCA CG and ACTCCAACTGCAAAGCTCCCTCAGC) and TNF.

Data were analyzed using ABI 7900HT machine (Applied Biosystems, USA). All primer sets were tested for specificity and equal efficiency before use.

Coimmunoprecipitation

(i) *Identifcation IRF5 domain that interacts with RelA*: HEK-293–TLR4-CD14/Md2 cells were co-transfected with ONEstrep tagged deletion IRF5 mutant or WT constructs and either FLAG tagged RelA or control Bacterial Alkaline Phosphatase (BAP). 24 hours post-transfection cells cells were lysed in RIPA buffer and immunoprecipitated on anti-FLAG M2 sepharose beads (Sigma). Flag peptide eluates were immunoblotted with anti-FLAG-HRP (A8952; Sigma) for bait or anti-HA-HRP (12013819001; Roche) for prey IRF5 proteins.

(ii) *Identification of RelA domain that interacts with IRF5*: HEK-293–TLR4-CD14/Md2 cells were co-transfected with ONEstrep tagged IRF5 WT construct and FLAG tagged deletion RelA mutants. 24 hours post-transfection cells were lysed in 1% TX-100 lysis buffer (containing) and affinity purified on Strep-Tactin Macrprep sepharose (IBA). Biotin eluates were immunoblotted with anti-Strep (IBA) for bait IRF5 and anti-FLAG –HRP (A8952; Sigma) for prey RelA proteins.

Protein Binding Microarrays (PBMs)

Sequences of the different primers and DNA ligands can be found in Additional file "primers_oligos_IRF". All quantification of nucleic acid samples was performed according to manufacturer instructions on a Qubit Fluorometer (Invitrogen #Q32857, Paisley, United Kingdom) and with either the Quant-iT dsDNA High Sensitivity Assay Kit (Invitrogen #Q33120) or the Quant-iT dsDNA Broad Range Assay Kit (Invitrogen #Q33130). Protein assays were performed using the Quant-iT™ Protein Assay Kit (Invitrogen #Q33210).

Protein expression and purification

Expression constructs for the IRF proteins (*Homo sapiens*) used in this study were created following a set of procedures previously established by Udalova and co-workers [42]. Briefly, pET vectors for expression in BL21 (DE3) *Escherichia coli* (Merck, Nottingham, United Kingdom) were used to produce

histidine-tagged (His-tagged) recombinant proteins. Proteins were over-expressed through induction with 0.2 mM isopropyl β-D-1-thiogalactopyranoside (IPTG) at 30°C for 5 hours. Pellets of cells were harvested in 'Ni-NTA binding' buffer with added EDTA-free protease inhibitor (Roche, West Sussex, United Kingdom), pulse-sonicated for 2 minutes and debris removed via centrifugation at 16,000 *g*. A two-step purification procedure was then employed, first with the 'Ni-NTA His-Bind Resin' system (Merck #70666) and then a subsequent purification based on DNA-affinity isolation of functional, DNA-binding protein. Ni-NTA purification was carried out according to the manufacturer's guidelines. For DNA-affinity isolation, the processing of a sample derived from 250 ml of bacteria culture required 0.128 µM of oligonucleotides specific for IRF protein binding. Prior to use, the oligonucleotides were annealed via incubation in NEB Buffer 3 at 94°C for 1 minute then subsequently for an additional 69 cycles of 1 minute each coupled to a per-cycle, step-wise decrease of 1°C. A pre-annealed oligo mixture (712.5 µl) was conjugated with streptavidin-agarose (Sigma, Dorset, United Kingdom) before once-purified material from the preceding step was added to it.

ChIP-Seq analysis

Reads were mapped onto mouse genome build 37 by NCBI and the Mouse Genome Consortium (Church et al., 2009) , downloaded from UCSC (Fujita et al., 2011), mm9) using bowtie 0.12.7 (Langmead et al., 2009)) with the following options:-n 2 -a --best --strata -m 1. Peaks were called with Zinba (version 2.02.01, (Rashid et al., 2011)) using default options, a window size of 200 and an FDR of 1%. Aligned reads and called peaks were visualized with IGV (Thorvaldsdottir et al., 2013). After visualization we noticed that abundant peaks in the 3' region of genes caused binding events in the 5' region of genes to go undetected. For the promoter analysis only we thus called peaks using MACS2 in a region of -10kb, 1kb around transcription start sites only. This added an additional 939 peaks in the Irf5 data set. Read densities were analyzed with in-house scripts.

Microarray analysis

Microarray (Accession number: E-MTAB-2032)data was analysed in R/bioconductor using the beadarray (version 2.4.2, Ref (Dunning et al., 2007))

and siggenes packages (version 1.28.0, Schwender 2012). Differentially expressed genes were called with SAM method (Ref (Tusher et al., 2001)) applying a false discovery rate threshold of 10%.

Interaction analysis and genomic enrichment

The significance of genomic enrichment was analysed using a simulation procedure similar to (Ponjavic et al., 2009). Briefly, the genomic association between a test set of peaks and a genomic annotation is measured by randomly simulating sets of peaks of equivalent size and length distribution to the test set. Enrichment and depletion are measured as ratio of the observed nucleotide overlap compared to the expected nucleotide overlap from 10,000 simulated sets and its significance is expressed as a P-Value. The significance of fold change difference is computed in an analogous manner by combining the results from two parallel simulations. Genomic regions of low mapability are excluded from the simulation. To control for biases in gene density, the overlap with chromatin marks was assessed in 50kb regions around genes only. For the overlap with transcription factors only regions 2kb upstream and 0.5kb downstream of transcription start sites were considered. The code for the simulations is publicly available (http://code.google.com/p/genomic-association-tester/).

Motif analysis

Motif analysis in ChIP-Seq peaks was performed using MEME-ChIP(Machanick and Bailey, 2011). Motif discovery was performed on the top 500 peaks using MEME-ChIP in 200 bp windows around the position with highest read density in a ChIP-Seq peak. Motif discovery used both repeat masked and unmasked sequence using the following options: "-dna -revcomp -mod anr -nmotifs 3 -minw 5 -maxw 30".

Protein binding microarrays

We designed 2 × 105K Agilent arrays using eArray (details given below). These arrays were comprised of two main sets of probes: 12-mer sequences designed for IRF binding and a set of 11-mer sequences design for NF-kB binding use for validation purposes. As an IRF consensus sequence, we used the motif NRWANNGARAVY that codes for a total of 3072 different motifs.

Experiments were carried out in technical replicates showing a 98% correlation. Z-scores were assigned to each sequence represented in the array. The sequences were ranked and used to produce binding motifs over all 12bp using weblogo (http://weblogo.berkeley.edu/)

Microarrays (PBMs)
Description of probe-design on the microarrays.
Our microarrays are chips of 2 arrays each with 104961 probes per array. Each array contains 1325 manufacturer-probes (Agilent) and 103636 customized probes. Each probe is represented using 4 different flanks of 4-nt length: AGCT, ATGA, AGTC, AGAT and each flanked probe is replicated 7 times. Additional "IRF_design_microarray.txt" shows a breakdown of the number and type of probes present on each array.

Protocol for generation and use of double-stranded protein microarrays.
Single stranded probes on each array were rendered double-stranded with the following procedure. For each array on a 2x150K chip, 820 µl of "ds-mix" (NEB buffer 2, 0.1 µM dsPrimer, 2.5 X BSA, 163 µM dNTPs, 1.63 µM of Cy3-dCTP and 27.2 U of Klenow DNA polymerase I) was dispensed onto a "1x205K gasket", combined with a chip, the entire unit sealed within a hybridization chamber and incubated within a rotating-oven at 37 ºC for 90 min. The following washes were then carried out: 6 washes in 0.01 % Triton-X/PBS for 3 min each followed by a 3 min wash in PBS. Arrays were dried via centrifugation. To ascertain overall success of the procedure, arrays were scanned using the Agilent Microarray Scanner at maximum power and the image analyzed for extent of Cy3-incorporation within individual probes.
Prior to hybridisation, arrays were blocked, washed according to manufacturer's guidelines and incubated in 2 % milk/PBS for 1 h at room temperature. This was followed by 2 washes (6 min each; 0.1% Tween-20/PBS followed by 0.01% Triton X-100/PBS) and ended with a brief rinse in water before drying via centrifugation. Hybridizations were performed using a protein concentration of 0.01 µg/µl in 420 µl of protein binding reaction mix (10 mM HEPES pH 8, 0.5 M NH_4OAC, 100 mM NaCl, 5 mM $MgCl_2$/MgAcetate, 1 mM DTT and 5% glycerol). Protein binding reaction mixes were dispensed into the different compartments of a 2x105K gasket slide (Agilent), combined with a chip and the entire unit sealed into a hybridization chamber. The assembled

unit was rotated in the hybridization oven for 1 h at room temperature. Arrays were then subsequently washed 6 times with 1 % Tween-20/PBS for 6 min each and a further 6 washes with 0.01 % Triton X-100/PBS for 6 min each. This was followed by a brief rinse in water and drying via centrifugation. Labelling of bound protein was carried out in two stages. Firstly, arrays were incubated with 0.8 µg of primary rabbit anti-His antibody (Santa Cruz) in a 2 % milk/PBS solution for 1 h at room temperature. This was followed by 6 washes with 0.05 % Tween-20/PBS for 3 min each and other 6 washes with 0.01 % Triton X-100/PBS for 3 min. Subsequently, arrays were incubated with 6 µg of secondary Cy5-conjugated anti-rabbit IgG antibody in a 2 % milk/PBS solution for 30 min at 37 °C before being washed as per above. Before drying, arrays were first rinsed in PBS for 6 mins and then briefly again in water.
Arrays were dried via centrifugation and scanned using the Agilent Microarray Scanner at maximum power.

Genotyping

The $IRF5^{-/-}$ line was genotyped for the DOCK2 mutation as described previously (Yasuda et al., 2013). Briefly, DNA was obtained from ear clips using REDExtract-N-Amp (Sigma) and PCR was performed using the following primers which detect the DOCK2 mutation as a 305-bp product: DOCK2In29.4F GAC CTT ATG AGG TGG AAC CAC AAC C; DOCK2InR22.3.1R GAT CCA AAG ATT CCC TAC AGC TCC AC. IRF5 mice possessing the mutation for DOCK2 were culled and all experiments were performed on a line that was wild-type for DOCK2.

Accession numbers:

Unprocessed data have been deposited at ArrayExpress under accession numbers E-MTAB-2031 (ChIP-Seq data), E-MTAB-2661 (ChIP-Seq data) and E-MTAB-2032 (microarray data)

SUPPLEMENTAL FIGURES

Figure S1:

(A) Cell surface receptor and cytokine expression in macrophages BMDMs differentiated with GM-CSF or M-CSF. FACS samples were collected at day 9 of differentiation and stained for F4/80, CD206 or MHCII, IRF5 after the cells were stimulated with LPS (100ng/mL; 4hrs) or left unstimulated. Data are representative of 6 experiments. **(B)** Scatter plots of RelA ChIP-seq peaks following LPS stimulation at 0.5 or 2hr with unstimulated condition. **(C)** Scatter plots of IRF5 ChIP-seq peaks following LPS stimulation at 0.5 or 2hr with unstimulated condition. **(D)** Specific Recruitment of IRF5 to example gene promoters (*Il6, ccl5, il12b, il1a, tnf, nfkbia, gadd45b, irf1, il12a* and *mllt6*) were analysed by qPCR in GM-BMDMs from either WT or IRF5 KO mice following LPS stimulation (100 ng/mL; 2hrs). No recruitment of IRF5 was observed on the negative control *Hbb* promoter. Data show mean percentage input relative to genomic DNA (gDNA) plus or minus SD of a representative experiment. **(E)** Average ChIP-seq enrichment profile of IRF5, RelA and Pol II binding regions around transcription start (TSS) and end site (TES) (dotted lines). IRF5, RelA and PolII show distinct binding to upstream of the TSS. IRF5 also displays binding downstream of the TES. **(F)** Binding of RelA, PolII and IRF5 in the 5' or 3' upstream regions of genes. The areas show the proportion of genes that have upstream binding only, downstream binding only, or both. Upstream regions are defined as 5kb upstream from the transcription start site. The downstream regions are similarly defined as 5kb downstream of the transcription termination site.

Figure S2:

(A) Percentage overlap of Pol II intervals with TSS of ENSEMBL gene set were categorised according to absence or presence of IRF5 and RelA peaks. 67% of PolII intervals that contain both a predicted IRF5 and RelA binding event overlap a TSS, while only 32% of PolII intervals without IRF5 and RelA binding overlap a TSS. **(B)** RelA and IRF5 alignment with PU.1 in promoters and enhancers: Intervals were classified according to chromatin marks H3K4Me3 and H3K4Me1 [14] into promoter-like (left panel) and enhancer-like (right panel) groups when the average density of H3K4Me3 > H3KMe1 and H3K4Me1 > H3K4Me3 respectively. Aggregate plots (normalized by total-max & counts) of H3K4Me1, H3K4Me3 and PU.1 (Garber et al., 2012) are centered on the RelA

peaks (Top plot: with IRF5; Middle plot without IRF5) and IRF5 peaks (Bottom plot: without RelA). FDR =1%.

Figure S3:

(A) PCR to detect the DOCK2 mutation. Genomic DNA from IRF5$^{-/-}$ (Left panel, Lanes 1-3) and IRF5$^{+/+}$ (Right panel, Lanes 7-9) did not result in a PCR product for the DOCK2 mutation (305bp; +ve controls Lanes 6 and 10). IRF5 was used as an internal control to verify the adequacy of DNA preparation in each sample (PCR products IRF5$^{-/-}$: 550 bp Lanes 1-3; IRF5$^{+/+}$: 650bp Lanes 7-9; IRF5$^{+/-}$: both 650 and 550 bp).

(B) Gene expression heatmaps of Category 1, 2 and 3 genes affected by IRF5 and RelA KO following LPS stimulation. GM-BMDMs from conventional IRF5 KO (left panel) or conditional RelA KO were each compared to WT controls following stimulation by LPS (100ng/mL) for 0,1,2,4 or 8 hrs; (Data are pooled from three experiments; blue to red represents increase level of gene expression).

Figure S4:

Identification of Consensus IRF Binding Motifs by custom IRF PBMs: Representative DNA-binding site motifs were determined for IRF5 using the total, top 500, top 275 and top 50 motifs bound. Data are derived from pooled data of two experiments.

Figure S5:

q-PCR analysis of IRF5-dependent genes and efficiency RelA knockdown by Cre virus: **(A)** GM-CSF BMDMs from IRF5-/- or WT mice were left stimulated or stimulated with LPS (100ng/mL) for 1,2,4,6 and 8h. *Il1a*, *Il6* and *Tnf* mRNA expression was compared to unstimulated WT control cells. Data shown are the mean ± SD of 4 independent experiments. **(B)** GM-CSF differentiated BMDMs from RelAFL mice were infected with adenoviral vectors encoding Cre or control (Empty) prior to stimulation with LPS (100ng/mL; 2hrs). Approximately 70% of RelA protein was degraded following Cre infection compared to empty control as analysed by Western blotting of RelA. IRF5 protein stability was unaffected following Cre infection

SUPPLEMENTARY TABLES

Table S1:

MappingChIP-seq reads to genome: **(A)** A total of 6 samples were analysed for ChIP-seq following LPS stimulation (100ng/mL) as indicated. For each sample the total number of reads sequenced (total) and the total reads mapped (mapped) are shown. Reads mapping to the exact same position (duplicates) were removed before peak calling. **(B)** The genome was segmented into annotated regions (cds, utr, upstream, downstream, intronic, intergenic) based on the ENSEMBL gene set. To avoid over-counting, an interval is associated with an annotation depending on the location of the peak (the point with the highest read density within an interval) **(C)** and **(D)** To assess whether IRF5 **(C)** and RelA **(D)** intervals are significantly associated with functional genome annotations (described in Figure1b), a simulation procedure was applied (*see Methods*). *Observed* (Observed nucleotide overlap between IRF5/RelA intervals and a genomic region); *Expected* (Expected nucleotide overlap between IRF5/RelA intervals and a genomic region based on simulations); *CI25low/CI95high* (95% confidence intervals); *Stddev* (Standard deviation of expected overlap); *Fold* (Fold change: Observed/Expected); *l2fold* (log2 fold change).

Table S2:

(**A**) Differentially expressed genes are called at FDR= 1% and having a greater than two-fold change in expression following LPS stimulation. **(B)** Fold enrichments of IRF5 and RelA ChIP-seq peaks at chromatin marked regions obtained by simulation procedure (*see Methods*) were used to assess whether IRF5 or RelA were associated with chromatin marks for enhancers (H3K4ME1) or promoters (H3K4ME3) in BMDMs (Barish et al., 2010) and BMDCs (Garber et al., 2012). All enrichments are statistically significant ($p<10^{-4}$) **(C)** Fold enrichments obtained by simulation procedure (*see Methods*) were used to assess degree of overlap of the IRF5:RelA cistrome with PU.1 or PU.1-less marked promoters or enhancers as indicated. All enrichments are statistically significant ($p<10^{-4}$).

Table S3:

Genes affected by conventional IRF5 KO **(A)** and conditional RelA KO **(B)** relative to WT following LPS stimulation split into categories as indicated in Figure 3A. GM-BMDMs from conventional IRF5 KO (left panel) or conditional RelA KO were each compared to WT controls following stimulation by LPS

(100ng/mL) for 0,1,2,4 or 8 hrs; (Data are pooled from three experiments; Down – expression decreased in KO. Up – expression increased in KO; bolding indicates genes affected in both IRF5 and RelA KO).

Barish, G.D., Yu, R.T., Karunasiri, M., Ocampo, C.B., Dixon, J., Benner, C., Dent, A.L., Tangirala, R.K., and Evans, R.M. (2010). Bcl-6 and NF-kappaB cistromes mediate opposing regulation of the innate immune response. Genes Dev *24*, 2760-2765.

Garber, M., Yosef, N., Goren, A., Raychowdhury, R., Thielke, A., Guttman, M., Robinson, J., Minie, B., Chevrier, N., Itzhaki, Z., *et al.* (2012). A high-throughput chromatin immunoprecipitation approach reveals principles of dynamic gene regulation in mammals. Mol Cell *47*, 810-822.

Yasuda, K., Nundel, K., Watkins, A.A., Dhawan, T., Bonegio, R.G., Ubellacker, J.M., Marshak-Rothstein, A., and Rifkin, I.R. (2013). Phenotype and function of B cells and dendritic cells from interferon regulatory factor 5-deficient mice with and without a mutation in DOCK2. International immunology *25*, 295-306.

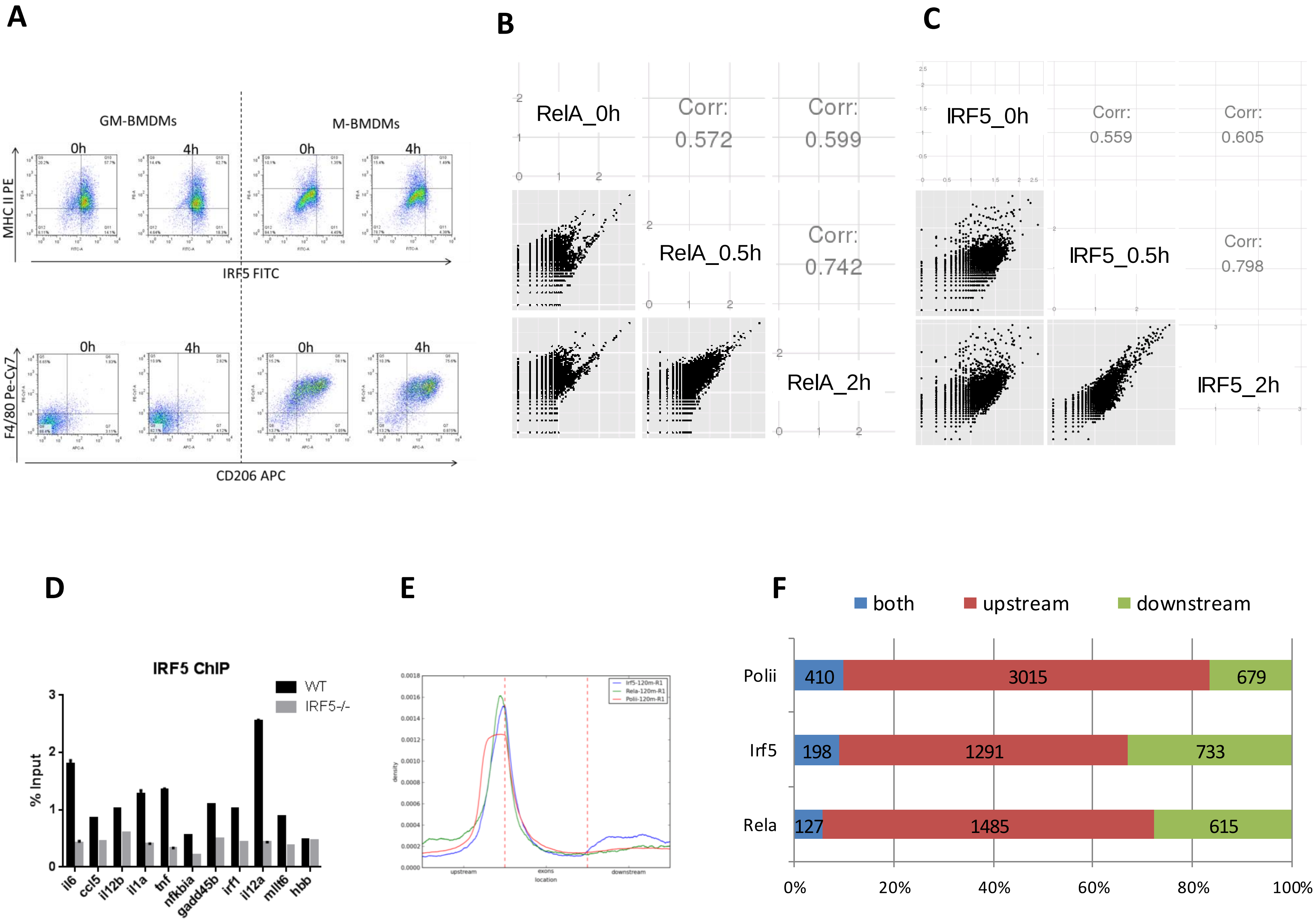

Supplementary Figure S1, related to Figure 1

A

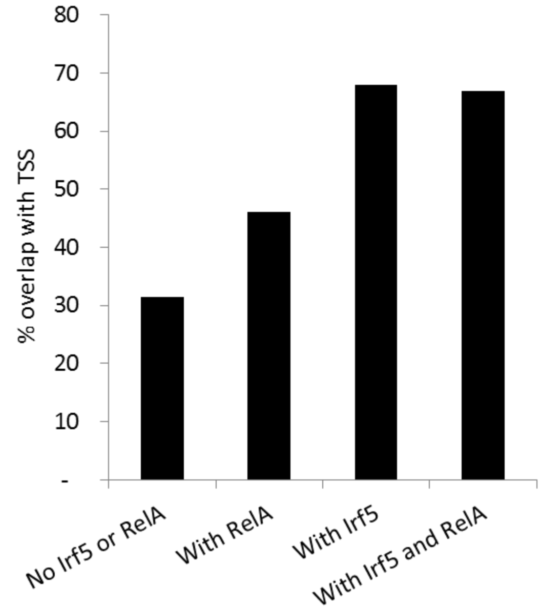

B

Promoter | Enhancer

RelA With Irf5

RelA Without Irf5

Irf5WithoutRelA

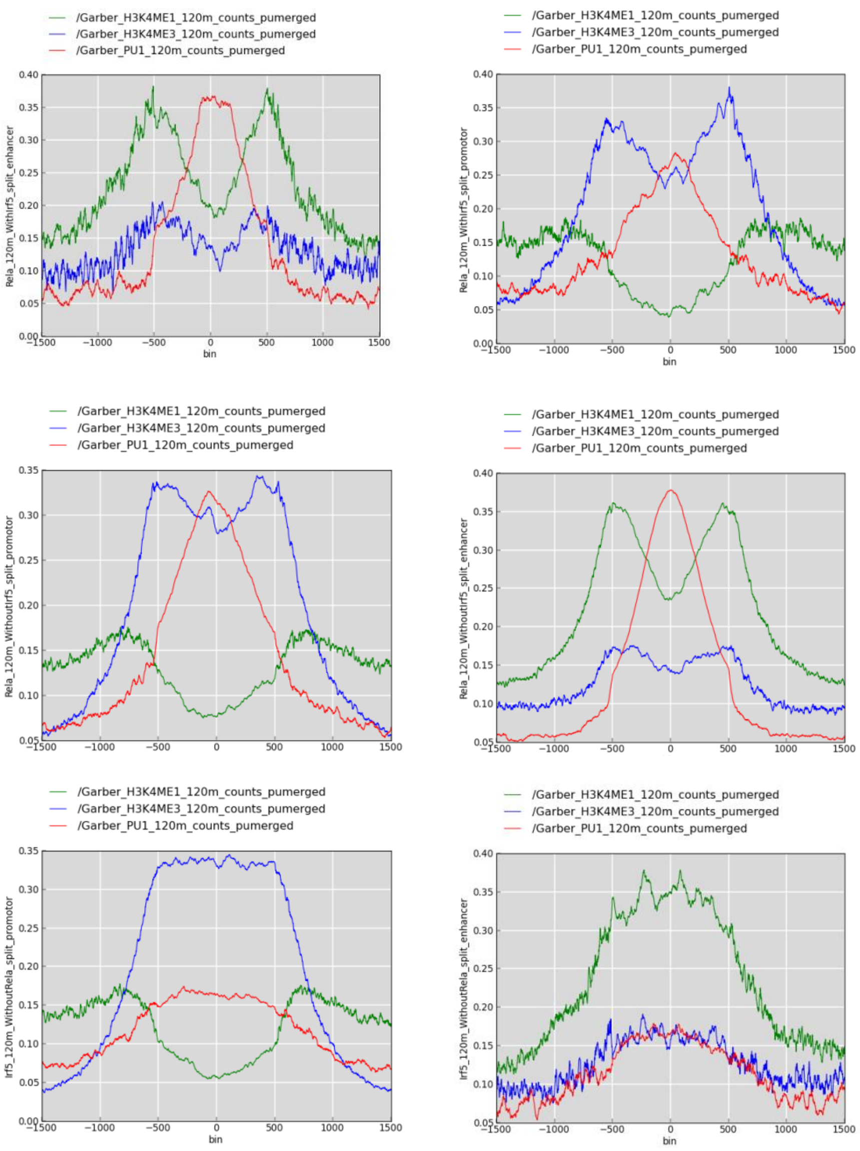

Supplementary Figure S2, related to Figure 2

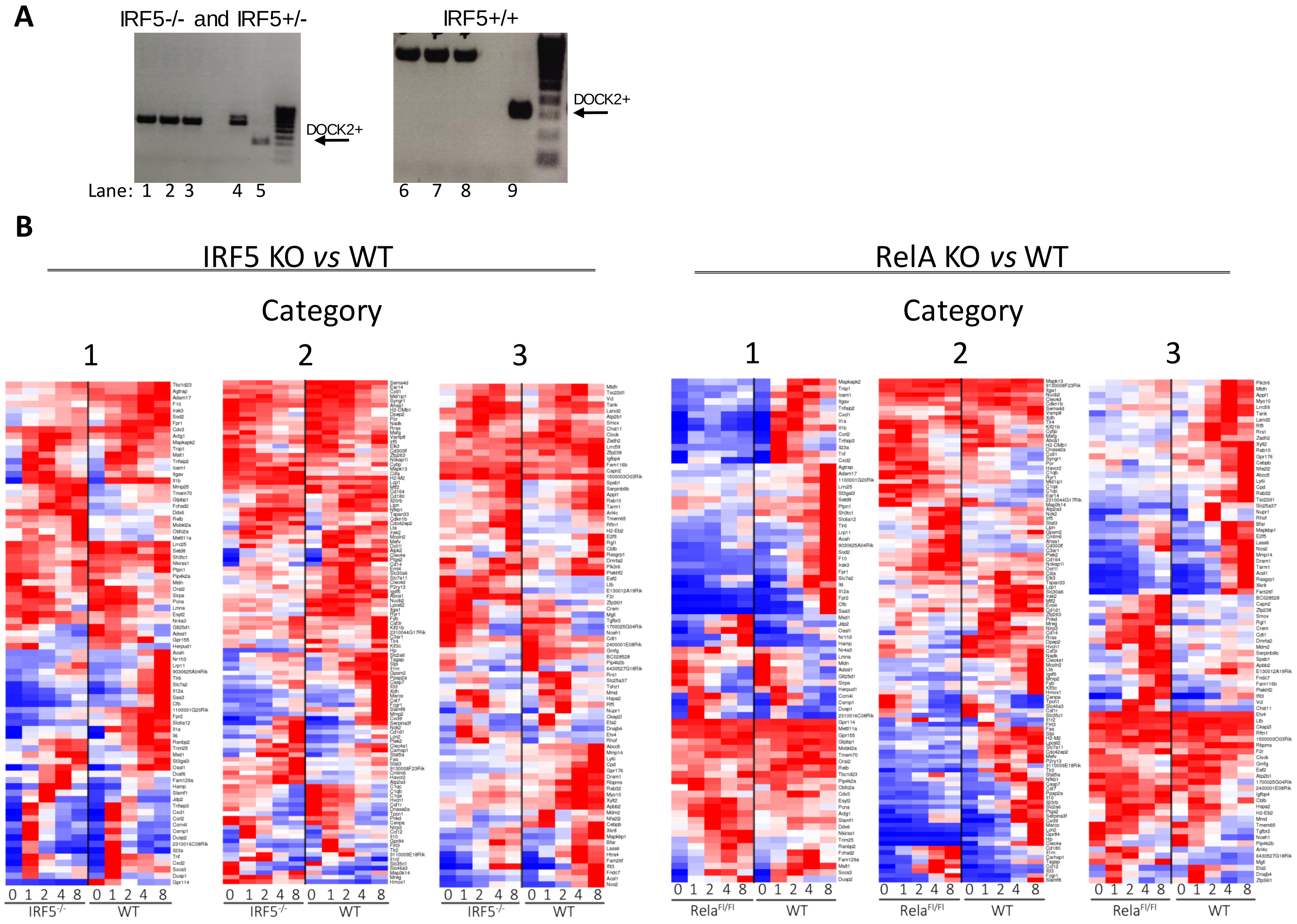

Supplementary Figure S3, related to Figure 3

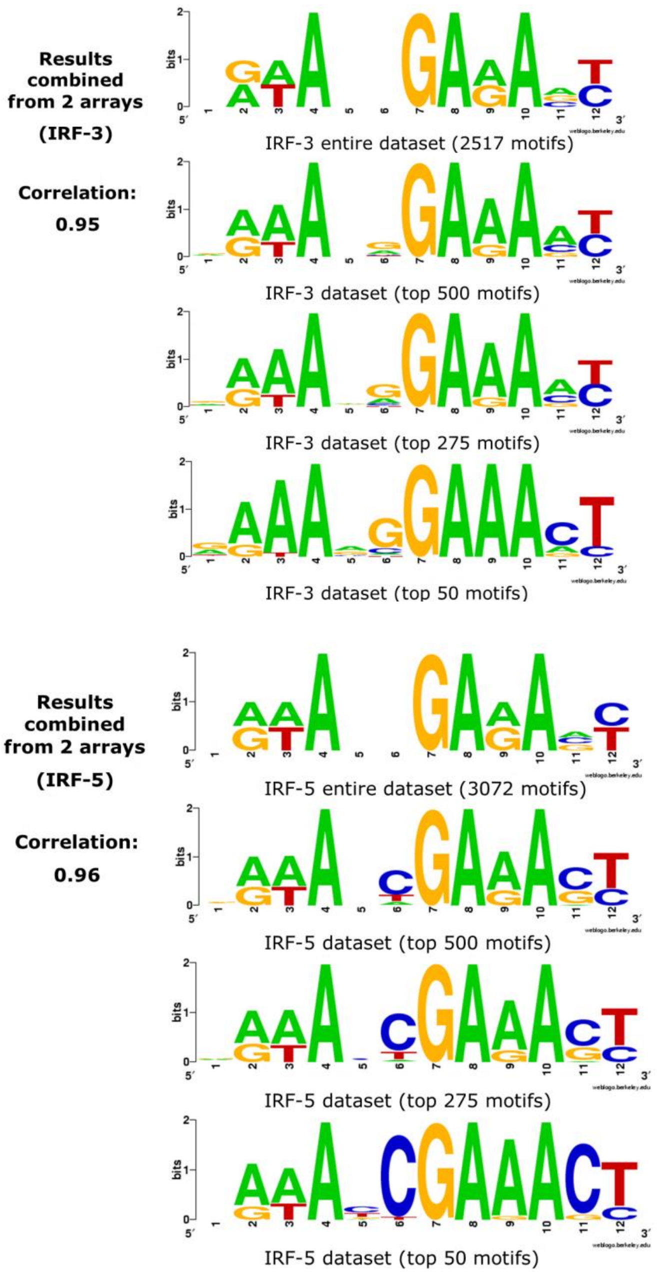

Supplementary Figure S4, related to Figure 4

A

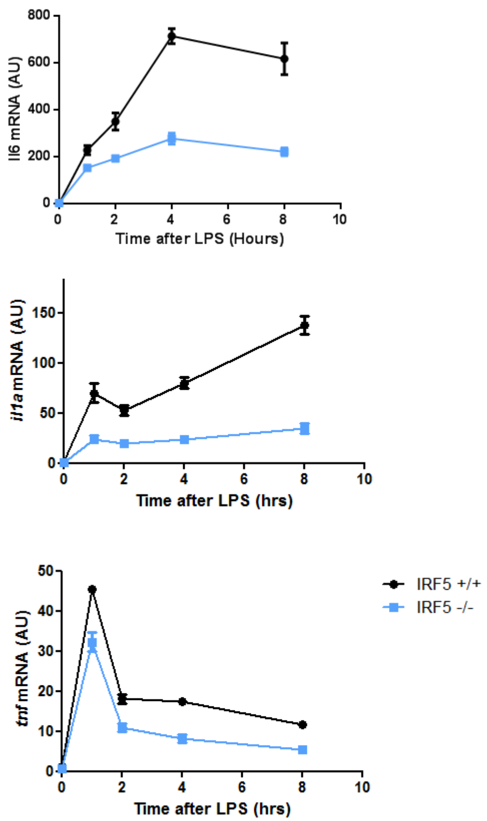

B

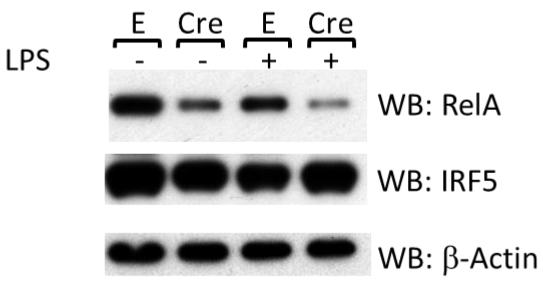

Supplementary Figure S5, related to Figure 5

Supplementary Table S1, related to Figure 1

A: number of sequences mapped to the genome

track	total	mapped	duplicates
Irf5/30'	22,921,738	17,486,718	4,352,213
Irf5/2h	11,006,735	7,928,653	1,174,331
Irf5/input	36,613,978	26,512,205	1,877,445
PolII/30'	2,372,589	1,437,092	108,247
PolII/2h	18,101,706	13,122,996	4,739,031
PolII/input	13,495,166	10,304,210	409,974
RelA/30'	8,722,397	5,235,902	334,544
RelA/2h	14,363,524	9,305,020	304,502
RelA/input	13,495,166	10,304,210	409,974

B: genome-wide distribution of mapped ChIP-Seq peaks

track	cds	utr	Up stream	Down stream	Intronic	Intergenic
IRF5/30'	57	58	119	194	1,583	2,103
IRF5/2h	281	508	490	480	1,264	970
PolII/2h	288	978	1,158	289	1,114	636
RelA/30'	146	584	1,073	241	1,965	1,684
RelA/2h	110	364	919	368	3,473	3,300

C: IRF5 ChIP-Seq interval nucleotide overlap with a genomic region 2h after LPS stimulation

annotation	observed	expected	CI95low	CI95high	stddev	fold	l2fold	p-value
intergenic	569,786	1,122,074	1,086,920	1,157,021	21116	0.5	-0.98	0.0001
intronic	854,072	1,209,453	1,174,133	1,244,637	21254	0.7	-0.50	0.0001
UTR3	64,216	42,083	34,133	50,265	4902	1.5	0.61	0.0001
CDS	147,997	59,050	52,683	65,651	3913	2.5	1.33	0.0001
upstream	329,704	105,740	92,234	119,578	8284	3.1	1.64	0.0001
downstream	330,675	96,689	83,460	110,237	8072	3.4	1.77	0.0001
UTR5	222,950	10,519	7,759	13,584	1768	21.2	4.41	0.0001

D: RelA ChIP-Seq interval nucleotide overlap with a genomic region 2h after LPS stimulation

annotation	observed	expected	CI95low	CI95high	stddev	fold	l2fold	pvalue
UTR3	58,864	73,239	63,548	83,260	5,984	0.8	-0.3	0.0063
CDS	82,724	101,229	93,341	109,260	4,846	0.8	-0.3	0.0001
intergen	1,730,230	2,017,149	1,975,502	2,059,215	25,629	0.9	-0.2	0.0001
intronic	1,903,324	2,126,694	2,085,354	2,169,435	25,622	0.9	-0.2	0.0001
downstream	197,311	168,714	153,064	184,640	9,539	1.2	0.2	0.0018
upstream	473,192	183,524	167,627	199,951	9,913	2.6	1.4	0.0001
UTR5	147,402	18,181	14,648	21,963	2,215	8.1	3.0	0.0001

Supplementary Table S2, related to Figure 2

A: IRF5 and RelA binding at promoters of strongly (>2-fold) up- and down-regulated genes. Differentially expressed genes are called at a false discovery rate of 1% and have a greater than two-fold change in expression.

**Category**	**Upregulated**	**Downregulated**	**Description**
IRF5+RelA	74	3	IRF5 and RelA present
RelA	65	12	Only RelA present
IRF5	53	30	Only IRF5 present

B: Fold enrichment of IRF5 and RelA ChIP-Seq peaks at chromatin marked regions

	**BMDM**		**BMDC**	
	**H3K4ME1**	**H3K4ME3**	**H3K4ME1**	**H3K4ME3**
**Irf5 (120')**	2.07	13.17	3.23	9.94
**RelA (120')**	8.20	7.53	5.48	6.31
**RelA and Irf5 (120')**	5.77	13.66	5.45	10.77

C: RelA:IRF5 binding at Pu.1 marked promoters and enhancers

	**H3K4ME1**	**H3K4ME3**
**All**	5.4	10.8
Pu.1	5.9	14.2
Without PU.1	1.5	5.6

A. Genes affected by IRF5 KO relative to WT following LPS stimulation

Category 1		Category 2		Category 3	
down	up	down	up	down	up
	Relb	**Slpi**	**Il1r2**	**Nfe2l2**	**Mgll**
**Aoah**	Mobkl2a	**Ppap2a**	Tspan33	**Acsl1**	Etv4
**Il1a**	Slamf1	**Csf1r**	Cdkn1b	**Ets2**	Atp2b1
**Slc6a12**	Mmp25	**Hp**	Stat5a	**Rasgrp1**	Zfp36l1
**1100001G20Rik**	Tmem70	**Mmp2**	Mapk13	**Gpr176**	Ltb
**Fpr2**	Obfc2a	**Gpr84**	Atp2a3	Cpd	Rhof
**Adam17**	Fam129a	**Hvcn1**	Map3k14	Ly6i	Eef2
**Cxcl2**	Dcaf6	Elk3	Plek2	Lass6	Rftn1
**Cfb**	Pip4k2a	Abca1	Ciita	Apbb2	E130012A19Rik
**Saa3**	Mettl11a	Xdh	Cd1d1	Rbpms	F2r
**Il12a**	Fchsd2	Gpsm2	Slc46a3	Htra4	Tmem65
Il23a	Ddx6	Clec4d	Cmtm6	BC028528	
Il6	Gtpbp1	Ryr1	Mreg	Fndc7	
Glt25d1		Nucb2	9130008F23Rik	Tshz1	
Gpr155		Tpcn1	Nck2	Ckap2l	
Socs3		Alpk2		Gmfg	
Gpr114		Tlr4		Nos2	
Adssl1		Itga1			
		Flrt3			
		Slamf8			

B. Genes affected by RelA KO relative to WT following LPS stimulation

Category 1		Category 2		Category 3	
down	up	down	up	down	up
**Irak3**	Trim25	**Slpi**	**Il1r2**	**Nfe2l2**	**Mgll**
**Aoah**	Mxd1	**Ppap2a**	Dnase2a	**Acsl1**	Mmd
**Il6**	Nr1h3	**Csf1r**	Stat3	**Ets2**	Cdt1
**Il1a**	Ranbp2	**Hp**	Por	**Rasgrp1**	1500003O03Rik
**1100001G20Rik**	Esyt2	**Mmp2**	Mid1ip1	**Gpr176**	Fam116b
**Fpr2**	Nkiras1	**Gpr84**	Carhsp1	Mmp14	Igfbp4
**Adam17**	Ccrn4l	**Hvcn1**	Fcgr1	Pip4k2b	Mdm2
**Cxcl2**	Dusp1	Hmox1	Cd164	Rab32	Serpinb9b
**Cfb**	Pcna	Mcoln2	Havcr2	Dram1	Vcl
**Saa3**	Dusp2	H2-M2	Ahsa1	Rab10	Cblb
**Il12a**	Lmna	Il10	Cd180	Rffl	Rgl1
Sod2	Nr4a3	Kif3c	Syngr1	Xylt2	Capn2
Mapkapk2	Csrnp1	Sema4d	Nckap1l	Lrrc59	Smox
Lrp11	Jdp2	Lcp1	Lipn	Tsc22d1	Nceh1
Tnfaip3	Midn	Fyb	Il33	Mtdh	Dnajb4
Tnip1	Oasl1	Zfp263	Cytip	Myo10	2400001E08Rik
Tnfaip2	2310016C08Rik	Mefv	Il1rn	Bfar	Tgfbr2
Tbc1d23	Hamp	Slc30a6	Cenpa	Abcc5	1700025G04Rik
Tnf	Actg1	Lta	Irf5	E2f5	6430527G18Rik
Itgav		Fas	Cotl1	Clock	Chst11
Il1b		Casp7	Tagap	Nupr1	BC028528
Ptpn1		Pnkd	2310044G17Rik	Mapkbp1	Spsb1
St3gal3		3110009E18Rik	Ccl12	Slc25a37	Dmrta2
Agtrap		Marco	C1qa	Xkr8	Arl4c
Cxcl1		Lcn2	C1qc	Appl1	Plekhf2
Slc6a12		Slc7a11	C1qb	Fam26f	Hspa2
F10		Tlr2	C3ar1	Pik3r6	Zfp238
Slc7a2		Nfkb1	Kif21b	Zadh2	Crem
Herpud1		Csf3r	Cd300lf	Tarm1	H2-Eb2
Malt1		Nadk	Ear14	Cebpb	Ifit3
Cdv3		Mtf2	Dot1l	Rrs1	
Sh3tc1		Cxcl9		Lancl2	
Icam1		Clec4e		Tank	
Sirpa		Ptgs2			
Orai2		Eml4			
Ccrl2		Nlrp3			
9030625A04Rik		Lpcat2			
Fpr1		Igsf6			
Setd8		Slc2a6			
Lrrc25		P2ry13			
Tlr6		Rras			
Relb		Il20rb			
		Cdc42ep2			
		Clec4a1			
		Slc35c1			
		Vamp8			
		Cd14			
		Mafg			
		Dpep2			
		Irak2			
		Serpina3f			
		Cst7			
		H2-DMb1			

Supplementary Table S3, related to Figure 3
