# Supplementary material for: IRF5:RelA Interaction Targets Inflammatory Genes in Macrophages
Source: Cell Rep. 2014 Aug 21;8(5):1308–17. doi: 10.1016/j.celrep.2014.07.034 (PMC4471814; doi:10.1016/j.celrep.2014.07.034)
Supplement: Document S2. Article plus Supplemental Information [file mmc2.pdf]

OPEN ACCESS
CellPress

Cell Reports
Report

IRF5:RelA Interaction Targets Inflammatory Genes in Macrophages

David G. Saliba,[1,5] Andreas Heger,[2,5] Hayley L. Eames,[1] Spyros Oikonomopoulos,[3] Ana Teixeira,[3] Katrina Blazek,[1] Ariadne Androulidaki,[4] Daniel Wong,[3] Fui G. Goh,[1] Miriam Weiss,[1] Adam Byrne,[1] Manolis Pasparakis,[4] Jiannis Ragoussis,[3] and Irina A. Udalova[1,*]
[1]Kennedy Institute of Rheumatology, University of Oxford, Roosevelt Drive, Oxford OX37FY, UK
[2]CGAT, MRC Functional Genomics Unit, University of Oxford, South Parks Road, Oxford OX13PT, UK
[3]Wellcome Trust Centre for Human Genetics, University of Oxford, Roosevelt Drive, Oxford OX3 7BN, UK
[4]Institute for Genetics, University of Cologne, Joseph-Stelzmann-Strasse 26, Cologne 50931, Germany
[5]Co-first author
*Correspondence: irina.udalova@kennedy.ox.ac.uk
http://dx.doi.org/10.1016/j.celrep.2014.07.034
This is an open access article under the CC BY license (http://creativecommons.org/licenses/by/3.0/).

SUMMARY

Interferon Regulatory Factor 5 (IRF5) plays a major role in setting up an inflammatory macrophage phenotype, but the molecular basis of its transcriptional activity is not fully understood. In this study, we conduct a comprehensive genome-wide analysis of IRF5 recruitment in macrophages stimulated with bacterial lipopolysaccharide and discover that IRF5 binds to regulatory elements of highly transcribed genes. Analysis of protein:DNA microarrays demonstrates that IRF5 recognizes the canonical IRF-binding (interferon-stimulated response element [ISRE]) motif in vitro. However, IRF5 binding in vivo appears to rely on its interactions with other proteins. IRF5 binds to a noncanonical composite PU.1:ISRE motif, and its recruitment is aided by RelA. Global gene expression analysis in macrophages deficient in IRF5 and RelA highlights the direct role of the RelA:IRF5 cistrome in regulation of a subset of key inflammatory genes. We map the RelA:IRF5 interaction domain and suggest that interfering with it would offer selective targeting of macrophage inflammatory activities.

INTRODUCTION

A finely tuned inflammatory response to microbial and endogenous insults is essential for host survival. During inflammation, gene programs are activated by orchestrated changes in transcription and are determined by transcription factors (TFs) binding to accessible DNA regulatory elements found in promoters and enhancers. Ubiquitously expressed TFs such as nuclear factor-κB (NF-κB), interferon regulatory factors (IRFs), and activator protein 1 (AP.1) each play a central role in eliciting an inflammatory response to extracellular Toll-like receptor 4 (TLR4) stimulation by lipopolysaccharide (LPS). These ubiquitous stimulus-inducible TFs appear to work in conjunction with lineage-restricted constitutive TFs, such as PU.1, to define lineage-specific enhancers (Ghisletti et al., 2010; Heinz et al., 2010). However, the regulatory logic underlying the activation of specialized gene expression programs is to a large extent unknown. TFs that respond to tissue-specific microenvironmental cues and fine-tune cellular identities (Ostuni and Natoli, 2011) add to the complexity of this regulatory logic.

In this context, we demonstrated that IRF5 is critical in establishing inflammatory phenotypes in vitro and is involved in the positive regulation of type 1 T helper (Th1)/Th17-associated mediators, such as interleukin-1 (IL-1), IL-12, IL-23, and tumor necrosis factor α (TNF-α) (Krausgruber et al., 2010, 2011). Moreover, IRF5 is capable of repressing anti-inflammatory genes associated with the macrophage colony-stimulating factor (M-CSF)-derived phenotype, such as *IL-10* (Krausgruber et al., 2011). A functional consequence of this dual role is demonstrated by studies showing that IRF5 is essential in the development of Th1 responses to *Leishmania donovani* infection (Paun et al., 2011) and in the susceptibility to lethal endotoxic shock (Takaoka et al., 2005). These divergent functions of IRF5 suggest that IRF5 cooperates with different cofactors at inflammatory versus homeostatic gene regulatory elements. In fact, we have reported that IRF5 forms a protein complex with NF-κB RelA to drive a sustained induction of the human *TNF* gene (Krausgruber et al., 2010).

In this study, we used GM-CSF (granulocyte/macrophage-colony-stimulating factor)-derived macrophages (GM-bone marrow-derived macrophages [BMDMs]) to investigate whether the recruitment of IRF5 via its interactions with RelA is a common mechanism of proinflammatory gene regulation by IRF5. By intersecting the chromatin immunoprecipitation-sequencing (ChIP-seq) analysis of the individual TFs in LPS-stimulated GM-BMDMs with gene expression data and histone methylation status data sets, we show that the IRF5 and RelA cistromes target inflammatory genes. The two cistromes overlap only at a limited number of genomic regions located in the PU.1-marked regulatory elements of inflammatory genes, 70% of which are induced upon LPS stimulation, as shown by the recruitment of RNA polymerase II (PolII). Using in vivo and in vitro motif discovery analyses, we demonstrate that the IRF5:RelA cistrome is best explained by the presence of consensus NF-κB and noncanonical composite PU.1:interferon-stimulated response element

(ISRE)-binding sites. We demonstrate that IRF5 genome recruitment to inflammatory genes is aided by RelA. These results reveal a genomic strategy for controlling an inflammatory gene program in GM-BMDMs via establishment of a unique IRF5:RelA cistrome to target inflammatory genes.

RESULTS

Genome-wide Alignment of IRF5 and RelA Binding in GM-CSF BMDMs

To investigate the model of IRF5-RelA transcriptional cooperation, ChIP-seq was used to determine the genome-wide binding of IRF5, RelA, and PolII in GM-BMDMs stimulated with LPS or left unstimulated. Upon LPS stimulations, these macrophages are predominantly homogeneous IRF5-positive cells that display a distinct phenotype of cytokine and cell surface molecule expression compared to M-CSF (CSF-1) (M)-BMDMs (Figure S1A) (Fleetwood et al., 2007; Weiss et al., 2013). ChIP-seq libraries were prepared for untreated cells or cells treated for 0.5 or 2 hr with LPS. Nonimmunoprecipitated input DNA isolated under the same conditions was also subjected to sequencing. Enriched bound genomic regions (peaks) were identified using the ZINBA (zero-inflated negative binomial algorithm; Rashid et al., 2011) at a false discovery rate (FDR) of 1% (Table S1A). We identified 1,252, 6,052, and 8,805 RelA peaks (RelA cistrome) and 3,591, 4,157, and 4,213 IRF5 peaks (IRF5 cistrome) at 0, 0.5, and 2 hr, respectively, post-LPS stimulation (Table S1B). The scatterplot analysis of the data sets demonstrated a strong influence of LPS stimulation on both RelA and IRF5 recruitment (Figures S1B and S1C). We also found an 80% overlap of RelA peaks identified in this study to peaks in GM-CSF-derived dendritic cells (GM-bone marrow dendritic cells [BMDCs]) (Garber et al., 2012). As a control for the IRF5 data set, we performed IRF5 ChIP quantitative PCR (ChIP-qPCR) in IRF5 knockout (KO) and wild-type (WT) cells stimulated with LPS and demonstrated a specific enrichment in IRF5 binding at the ChIP-seq-identified peaks in WT cells (Figure S1C). We also identified 1,679 and 4,879 PolII peaks 0.5 and 2 hr, respectively, post-LPS stimulation.

Some illustrative binding regions are shown in Figure 1A, including the lymphotoxin-α (*Lta*), *Ltb*, and *Tnf* gene cluster, chemokine (C-C motif) ligand 5 (*Ccl5*), IL-1α (*Il-1a*), and other immune related gene loci. In the case of the *Tnf* gene cluster, IRF5 and RelA show similar binding patterns as we previously reported in human monocyte-derived dendritic cells (Krausgruber et al., 2010). The peaks called by the ZINBA in these representative regions indicate considerable overlap of IRF5 and RelA binding to this gene locus. In general, IRF5 binding was found to co-occur frequently with RelA binding (801 peaks at 1% FDR) (Figure 1B). Using a simulation procedure that controls for genomic background (Ponjavic et al., 2009), we observe that the overlap between RelA- and IRF5-binding sites is 3.4-fold greater than expected ($p < 10^{-4}$). Therefore, the overlap of IRF5 and RelA binding initially observed in the *Tnf* gene cluster (Krausgruber et al., 2010) (Figure 1A) reflects a genome-wide phenomenon. Of interest, a significant enrichment in co-occurrence of RelA peaks with another member of the IRF family, IRF1, but not with IRF2 or IRF4, was observed in a high-throughput analysis of TF binding in GM-BMDCs (Garber et al., 2012).

Using the simulation procedure described above, we observed a 21-fold ($p < 10^{-4}$) increase in IRF5 recruitment following LPS stimulation for 2 hr (Table S1C). IRF5 peaks were also observed downstream of the 3′ UTRs of protein-coding genes (Table S1C; Figure S1D), with 33% of IRF5-targeted genes containing an IRF5 peak downstream of the transcription end site (Figure S1E). Binding of RelA upstream of transcription start sites (TSSs) amounted to 19% of all peaks at 2 hr post-LPS stimulation, but no significant enrichment in binding to intergenic, intronic, and downstream regions was observed (Table S1D). Correspondingly, the proportion of RelA peaks that co-occur with IRF5 peaks (IRF5:RelA cistrome) is markedly increased in the proximal 1 kb regions upstream of the TSS (5.2-fold change; $p < 0.0001$; Figure 1C). In summary, these analyses highlight that up to 20% of IRF5- and RelA-binding events occur within a limited part of the genome, namely the relatively short regions just upstream of protein-coding genes.

IRF5 and RelA Cistromes Intersect at PU.1-Marked Regulatory Elements of LPS-Induced Genes

To understand whether the binding of IRF5 and RelA influences PolII recruitment, we analyzed the degree of genome-wide overlap between IRF5 and RelA with PolII occupancy following 2 hr of LPS stimulation. PolII peaks overlapped with the IRF5:RelA cistrome (>116-fold over genomic background; $p < 10^{-4}$) more prominently than with the rest of either RelA or IRF5 peaks (Figure 2A). Analysis of the degree of PolII overlap with TSSs demonstrated increased overlap with TSSs when both IRF5 and RelA bind the gene upstream region (Figure S2A). Moreover, when we combined IRF5- and RelA-binding data with microarray gene expression data at the same time point, we noted that the peaks of the IRF5:RelA cistrome were centered around the TSS of strongly upregulated genes, whereas the RelA peaks that did not overlap with IRF5 displayed more uniform distribution in both upregulated and downregulated genes around the TSS (Figure 2B). Further analysis of gene expression across the stratified ChIP-seq peaks revealed that genes targeted by both RelA and IRF5 were significantly more upregulated than either RelA ($p < 10^{-7}$) or IRF5 ($p < 10^{-12}$) acting independently (Figure 2C). Among 340 strongly (>2-fold; FDR, 1%) upregulated genes, 74 were targeted by both RelA and IRF5 (Table S2C). RelA binding explains a similar number of upregulated genes, whereas IRF5 explains fewer. Of interest, the individual presence of IRF5 at the gene promoter explains a much larger proportion of 202 strongly downregulated (>2-fold; FDR, 1%) genes (Table S2A).

Next, we examined whether the RelA and IRF5 cistromes show characteristic chromatin signatures of functional genomic elements, i.e., enhancers and promoters marked by relatively high levels of monomethylation or trimethylation of lysine 4 of histone 3 (H3K4), respectively. To do this, we intersected the data sets with H3K4me1/H3K4me3-positive regions from M-BMDMs (Heinz et al., 2010) and GM-BMDCs (Garber et al., 2012). IRF5 and RelA peaks associated with both H3K4Me1 and H3K4Me3 chromatin marks ($p < 10^{-4}$; Table S2B).

It has recently been shown that binding of a pioneer TF PU.1 is essential for defining macrophage-specific enhancers because it promotes the deposition of H3K4me1 (Ghisletti et al., 2010;

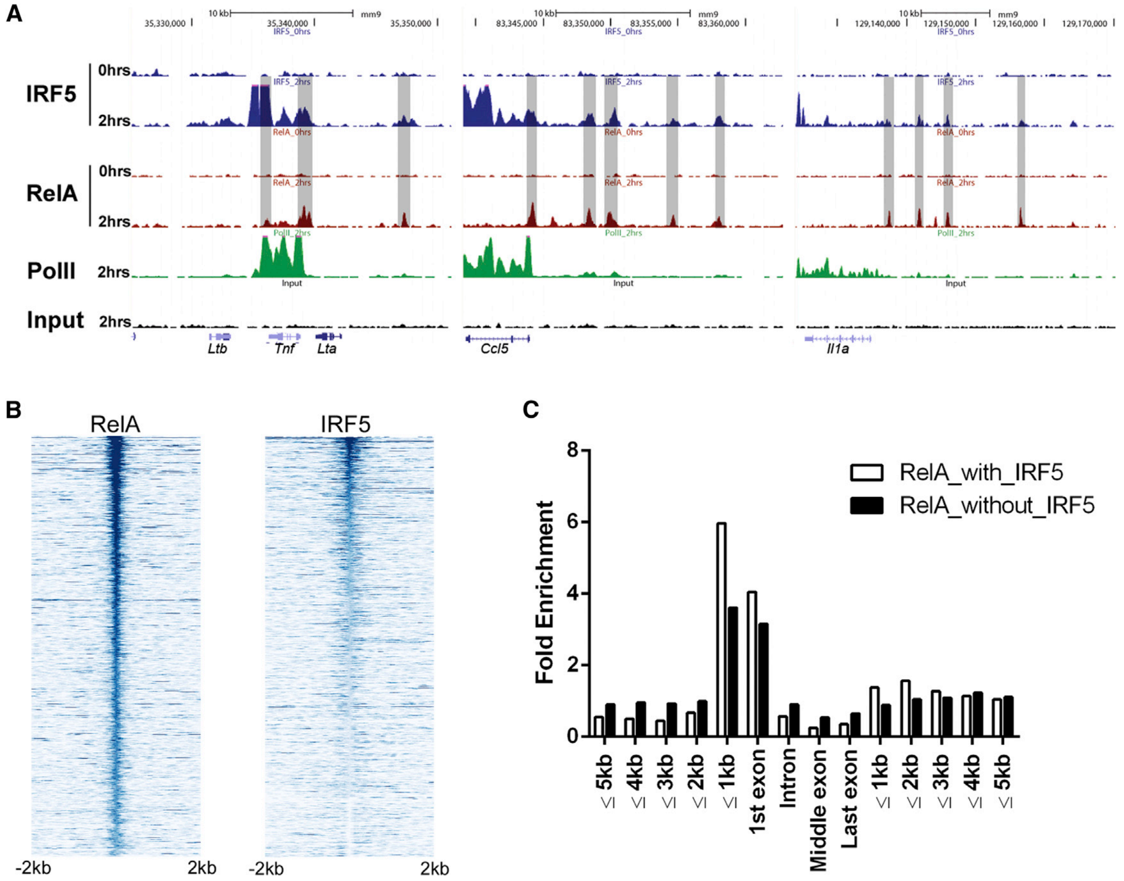

Figure 1. Distribution of IRF5- and RelA-Bound Regions in the Genome following LPS Stimulation

(A) Representative USCS Genome Browser tracks in the *Tnf*, *Ccl5*, and *Il-1a* loci for IRF5 (blue), RelA (red), PolII (green), and Input (black) of unstimulated (0 hr) or LPS-stimulated (2 hr) GM-BMDMs. Overlapping IRF5 and RelA regions are highlighted in gray.

(B) IRF5 reads colocalize with RelA peaks. The ChIP-seq data sets for RelA and IRF5 (±2 kb) were each aligned with respect to the center of the RelA peaks and sorted by the height of the RelA-marked regions. Each line represents a RelA peak. A total of 1,000 representative peaks are shown.

(C) Fold enrichment of RelA ChIP-seq peaks with (white bars) or without (black bars) IRF5 was aligned to the nearest gene structures in which 5 kb upstream/downstream regions were split into 1 kb windows. A 5.2-fold enrichment of overlapping RelA and IRF5 peaks at the proximal 1 kb region to 3.7-fold for RelA peaks that do not co-occur with IRF5 ($p = 10^{-4}$, as defined by simulation procedure; see Experimental Procedures).

See also Figure S1 and Table S1.

Heinz et al., 2010). Here, we examined the aggregated densities of PU.1 reads reported in Garber et al. (2012) over IRF5:RelA peaks and found that they align perfectly with the peaks of H3K4me1 and H3K4me3 deposition at enhancers and promoters, respectively, with the notable bimodal distribution of histone marks indicative of nucleosome depletion (Figure S2A). Moreover, binding of IRF5:RelA peaks occurred at both PU.1-marked promoters and enhancers ($p < 10^{-4}$; Table S2C). Thus, the genes that expression is strongly induced in macrophages by LPS are under the control of the IRF5:RelA cistrome, which is centered around the TSS.

The IRF5:RelA Cistrome Targets Regulatory Elements of Key Inflammatory Genes

To identify genes that are directly and functionally affected by either the IRF5:RelA cistrome or IRF5 and RelA acting individually, we first categorized promoters (up to 10 kb upstream and 0.5 kb of the TSS) of the genes into three categories. These consist of genes that encompass ChIP-seq peaks present in (1) both IRF5 and RelA, (2) only RelA, or (3) only IRF5 (Figure 3A). We next performed global expression profiling to identify LPS-affected genes that are differentially expressed in either GM-BMDMs from IRF5 conventional KO or conditional RelA KO (RelA Fl/Fl Mx1Cre) (Luedde et al., 2008) compared to WT mice (Figure 3B). The expression profiles of GM-BMDMs at 0, 1, 2, 4, and 8 hr after LPS stimulation were analyzed for differential gene expression. IRF5 or RelA deficiency resulted in inhibition of a very selective subset of category 1 genes that encompassed key inflammatory mediators (defined as Panther Pathway "Inflammation mediated by chemokine and cytokine signaling pathway"; Hyper FDR, $q < 10^{-10}$), such as *Il-6*, *Il-12a*, *Il-1a*, *Fpr2*, *Aoah*, *Adam17*, *Cxcl2*, and *Saa3* (Figure 3C). Moreover, expression of some other important inflammatory genes was affected by either only IRF5 deletion (*Mmp25* and *Socs3*)

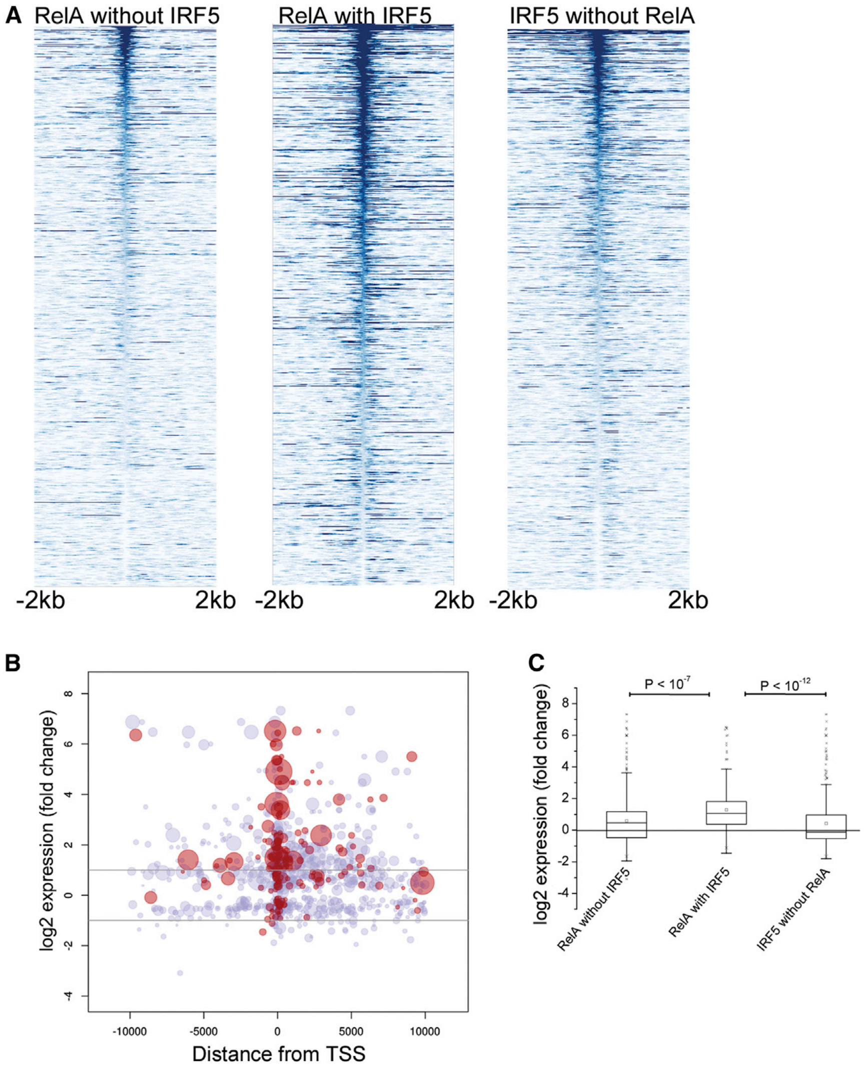

Figure 2. IRF5 and RelA Colocalize at TSSs of Positively Regulated Genes

(A) IRF5 and RelA complex colocalizes with PolII peaks. The ChIP-seq reads for RelA without IRF5, RelA with IRF5, and IRF5 without RelA were each aligned with respect to the center of the PolII peaks and sorted by the height of the PolII-marked regions. A total of 500 representative peaks are shown.

(B) Bubble plot representation of RelA-binding sites around differentially regulated genes. The plots indicate the position of ChIP-seq peaks with respect to the closest TSS (x axis) and the observed fold change (y axis) in microarray expression experiments. The size of a bubble denotes the strength of the ChIP-seq peak. Red bubbles indicate RelA with IRF5; blue bubbles indicate RelA without IRF5.

(C) Box plot of gene expression fold change differences upon LPS stimulation with three categories of binding events in their vicinity as indicated. The fold change differences are significant (Mann-Whitney U test).

See also Figure S2 and Table S2.

or only RelA (e.g., *Tnfaip3*, *Itgav*, *Malt1*, *Icam1*, and *Sod2*) (Figure 3C). In category 2 genes, we observe that RelA, but not IRF5, KO has a direct effect on expression at, for example, *Lcn2*, *Fas*, *H2-M2*, *Clec4a1*, *Casp7*, and *Nlrp3* gene loci (Figure S3; Table S3). In category 3 genes, we observe that the lack of IRF5, but not RelA, has affected expression of, for example, *Nos2*, a key marker of M1 activity (Figure S3; Table S3). The observation that the expression of some genes in category 2 is affected by IRF5 KO and some genes in category 3 by RelA KO indicates an indirect effect via a secondary regulator in a feedforward loop (Mangan and Alon, 2003). In total, we find that 263 and 499 transcriptionally active genes are affected by the lack of IRF5 or RelA, respectively. Consistent with the published data demonstrating that most RelA target sites were not associated with transcriptional changes (Lim et al., 2007), at many loci, IRF5 or RelA binding did not directly correlate with change in gene expression, even in the absence of a TF. It is possible that previously noted "billboard" organization of immune genes' promoters (Garber et al., 2012) allows for a degree of redundant recruitment of TFs. Hence, the combined global profiling of IRF5- and RelA-bound sites and gene expression in cells deficient in either TF has highlighted the direct role of the IRF5:RelA cistrome in transcriptional regulation of selective key inflammatory genes.

The Presence of Canonical κB and Composite PU.1:ISRE Sites Is Characteristic of the IRF5:RelA Cistrome

To better understand the regulatory code at inflammatory loci, we first performed ab initio DNA motif analysis around the top 500 RelA and IRF5 peaks. Motif analysis revealed the known κB motif (Natoli et al., 2011; Siggers et al., 2012) in RelA peaks (Figure 4A). The same analysis of IRF5 peaks found no motifs similar to known canonical ISRE, A/GNGAAANNGAAACT (Badis et al., 2009; Tamura et al., 2008) (Figure 4A). However, the PU.1-binding motif was the top-scoring motif enriched in the IRF5 peaks (Figure 4A). The other top-binding motif in this data set included TFs recognizing CpG-rich sequences that are associated with gene promoters, such as Sp1 (Figure 4A). Interestingly, the binding regions co-occupied by IRF5 and RelA were enriched in κB site and binding motif that resembled a composite PU.1:ISRE, with the PU.1 site (Figure 4A, boxed) adjacent to the ISRE half-site (Figure 4A). This site was previously reported for immune cell development-related IRF4 and IRF8 (Brass et al., 1996; Escalante et al., 2002; Tamura et al., 2005). Additionally, whereas RelA binds to the κB site in the absence of IRF5, the latter is likely to bind to the CpG-rich sequence SP1 in the absence of RelA (Figure 4A). Thus, the mode of IRF5 in vivo binding groups it with immune cell development-related IRFs and strongly suggests that such IRF proteins exert their function as cofactors and not individually.

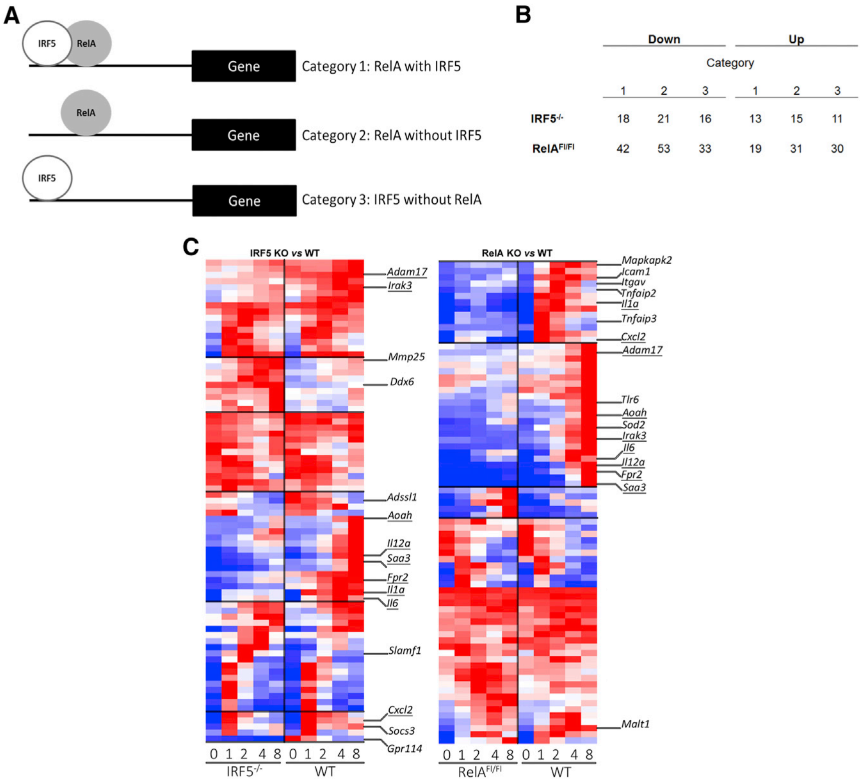

Figure 3. Association of IRF5:RelA-Bound Genes with Immune Genes

(A) Schematic of promoters bound by IRF5 and RelA. Each promoter (10 kb upstream and 0.5 kb of the TSS) containing IRF5 and/or RelA ChIP-seq peaks was classified into (1) bound by both IRF5 and RelA, (2) bound by RelA but not IRF5, or (3) bound by IRF5 but not RelA.

(B) Genome-wide profiling of LPS-affected genes in GM-BMDMs from either conventional IRF5 KO or conditional RelA KO (RelA Fl/Fl Mx1Cre; in which Mx1-Cre expression was induced by PolyI:C) compared to WT. Number of genes in each category significantly affected by KO of IRF5 or RelA (>2-fold; FDR, 1%) is shown (Down, regulator induces gene; Up, regulator represses gene). IRF5 KO resulted in 164 downregulated genes and 99 upregulated genes compared to WT. RelA KO resulted in 263 downregulated genes and 236 upregulated genes compared to WT.

(C) Gene expression heatmaps of category 1 genes. GM-BMDMs from conventional IRF5 KO (left panel) or conditional RelA KO each compared to WT controls following stimulation by LPS for 0, 1, 2, 4, or 8 hr are shown; underlining indicates genes that are affected in both IRF5 and RelA KO. (Data are pooled from three experiments; blue to red represents increase level of gene expression.) See also Figure S3 and Table S3.

Because ChIP only identifies genomic regions that interact with TFs but not necessarily individual binding sites (Gordân et al., 2009; Jolma et al., 2010; Wong et al., 2011), we used protein-binding microarrays (PBMs) for purified recombinant IRF3 and IRF5 protein to map the site of TF-DNA interactions with precision. For comparison, PBM data for RelAp50 were used from Wong et al. (2011). We analyzed 3,072 12-mer sequences designed around the ISRE consensus (see Experimental Procedures) carrying 4 different flanks. The sequences were ranked and used to produce binding motifs. The logo emerging from the top 50 binders was very similar to the one obtained by Badis et al. (2009), whereas the top 500 sequences produced a motif that was less stringent in positions 6, 9, and 11 (Figure S4, bottom panel).

All the IRF5- and RelAp50-binding sequences with their respective *Z* scores were used to perform receiver operating characteristic (ROC) curve analyses using the IRF5 and RelA cistromes from this study to quantify whether the IRF5- or RelA-bound regions (true positives) scored higher than the unbound regions (true negatives), similar to Siggers et al. (2012). We observed equally large areas under the ROC curve (AUC) when RelAp50 kmers were used to explain the IRF5:RelA cistrome peaks (AUC, 0.68) or the rest of RelA peaks (AUC, 0.65) compared to true negatives (Figure 4B). Thus, we concluded that RelAp50 binds to sequences in vivo that resemble its DNA-binding preferences in vitro. In contrast, AUC enrichment scores for IRF5 kmers demonstrated low nondiscriminating enrichment scores for the peaks of the IRF5:RelA cistrome (AUC, 0.53) and the rest of IRF5 peaks (AUC, 0.50) (Figure 4C).

Together with the ab initio analysis above, we therefore interpret this result as evidence that at the inflammatory gene loci, IRF5 is likely to be recruited to a composite PU.1:ISRE site rather than to a canonical ISRE site, whereas RelA binds directly to the respective κB site. The dynamics of PU.1 and IRF5 binding at the PU.1:ISRE composite sites are unclear and warrant further investigation.

RelA Aids in IRF5 Recruitment to Promoters of Inflammatory Genes

We previously reported that IRF5 can functionally interact with RelA at the human TNF locus (Krausgruber et al., 2010). Here, we addressed the question whether recruitment of IRF5 to inflammatory gene loci is commonly mediated by RelA. We examined IRF5 recruitment to the selected genes, expression of which was shown to be directly dependent on IRF5 (Figures 3B and S5A) in cells with depleted levels of RelA. GM-BMDMs were generated from the bone marrow of RelAFl/Fl mice (Luedde et al., 2008) and infected with Cre-expressing adenovirus. Efficiency of RelA deletion was about 50% as judged by analysis of residual RelA protein by western blot (Figure S5B). Following stimulation with LPS for 2 hr, recruitment of IRF5, as well as RelA and PolII, to the *Il-1a*, *Il-6*, and *Tnf* genomic loci was analyzed by ChIP-qPCR. We observed a significant reduction in IRF5 binding to the regions of overlapping IRF5:RelA peaks (Figure 5A). Thus, together with the previously observed dependence of IRF5 binding to the 3′ region of the human TNF gene (Krausgruber et al., 2010), our results suggest that recruitment of IRF5 to DNA at inflammatory gene loci is assisted by RelA.

OPEN ACCESS
CellPress

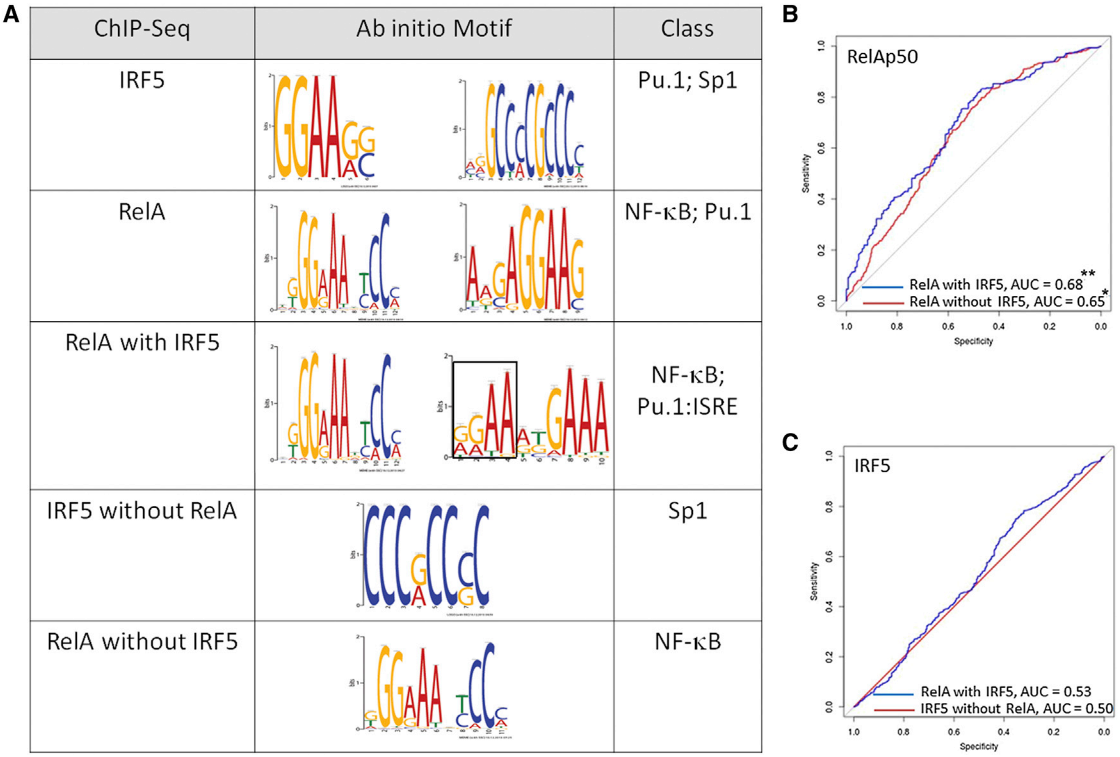

Figure 4. Enrichment of PBM-Determined κB Sites, but Not ISREs, in ChIP-Seq Peaks

(A) A parallel version of Multiple EM for Motif Elicitation-ChIP (Machanick and Bailey, 2011) was used to perform a de novo sequence search in the five subtypes of ChIP binding using 500 top-binding sequences in each category. The PU.1 motif (GGAA) and Sp1 motif were derived for the entire IRF5 data set. The κB and PU.1 motifs were derived for the entire RelA data set. The κB and composite PU.1 (boxed):ISRE motifs were derived for overlapping RelA and IRF5 peaks. The Sp1 motif was derived for the IRF5 data set in the absence of RelA, and the κB site was derived for the RelA data set in the absence of IRF5. (All motifs shown have an e value of $<10^{-5}$.)

(B) ROC curve analysis quantifying enrichment within RelA-bound regions (blue indicates with IRF5; red indicates without IRF5) of RelA-p50 PBM-determined sites.

(C) ROC curve analysis quantifying enrichment within IRF5-bound regions (blue indicates with RELA; red indicates without RelA) of IRF5 PBM-determined ISREs. AUC values quantify enrichment. (Wilcoxon-Mann-Whitney U test, $^{**}p < 10^{-29}$; $^{*}p < 10^{-7}$.)

See also Figure S4.

To map the interacting domains, we generated in-frame One-STrEP and HA-tagged truncation mutants of the key domains of IRF5 (Figure 5B, top panel) and FLAG-tagged truncation mutants of the key domains of RelA (Figure 5C, top panel). IRF5 truncation mutants or p50 (as positive control) was coexpressed to equal levels in HEK293-TLR4-CD14/Md2 cells along with RelA-Flag or BAP-Flag as negative control. The resulting lysates were subjected to One-STrEP immunoprecipitation, and the precipitated proteins were analyzed by immunoblotting with anti-HA antibody. The truncation mutants IRF5-ΔN219 and IRF5-N395 were comparable to the WT protein in binding RelA-Flag. In contrast, removal of the IRF association domain (IAD) in truncation mutants IRF5-N130 and IRF5-N220 resulted in impaired binding to RelA-Flag (Figure 5B, bottom panel). Flag-tagged RelA truncation mutants or BAP-Flag as negative control was coexpressed to similar levels along with One-STrEP-IRF5, subjected to One-STrEP immunoprecipitation, and the precipitated proteins were analyzed by immunoblotting with anti-FLAG antibody. The removal of the dimerization domain (DD) in truncation mutant RelA-N186 resulted in impaired binding to One-STrEP-IRF5 (Figure 5C, bottom panel). Thus, IRF5 IAD and RelA DDs are critical for IRF5-RelA interactions.

DISCUSSION

An emerging view on the transcriptional networks that dictate the response of macrophages while encountering microbial stimuli is that they consist of pioneer lineage-specific (e.g., CEBPβ, PU.1), basal (e.g., JunB, ATF3), and stimulus-inducible (NF-κB, IRFs, AP.1) TFs (Garber et al., 2012). Here, we investigated how TFs that define functional macrophage specialization (Garber et al., 2012; Ghisletti et al., 2010; Heinz et al., 2010), such as IRF5, contribute to the determination or regulation of specific subsets of the regulatory elements. We demonstrate that IRF5 is recruited to such elements of LPS-induced inflammatory genes and is essential for their efficient transcription. We find that NF-κB RelA assists IRF5 in binding to DNA, and the two factors set up a unique "inflammatory" IRF5:RelA cistrome. We also map the interface of IRF5:RelA interactions, paving the way to possible new therapeutics, that would specifically reduce

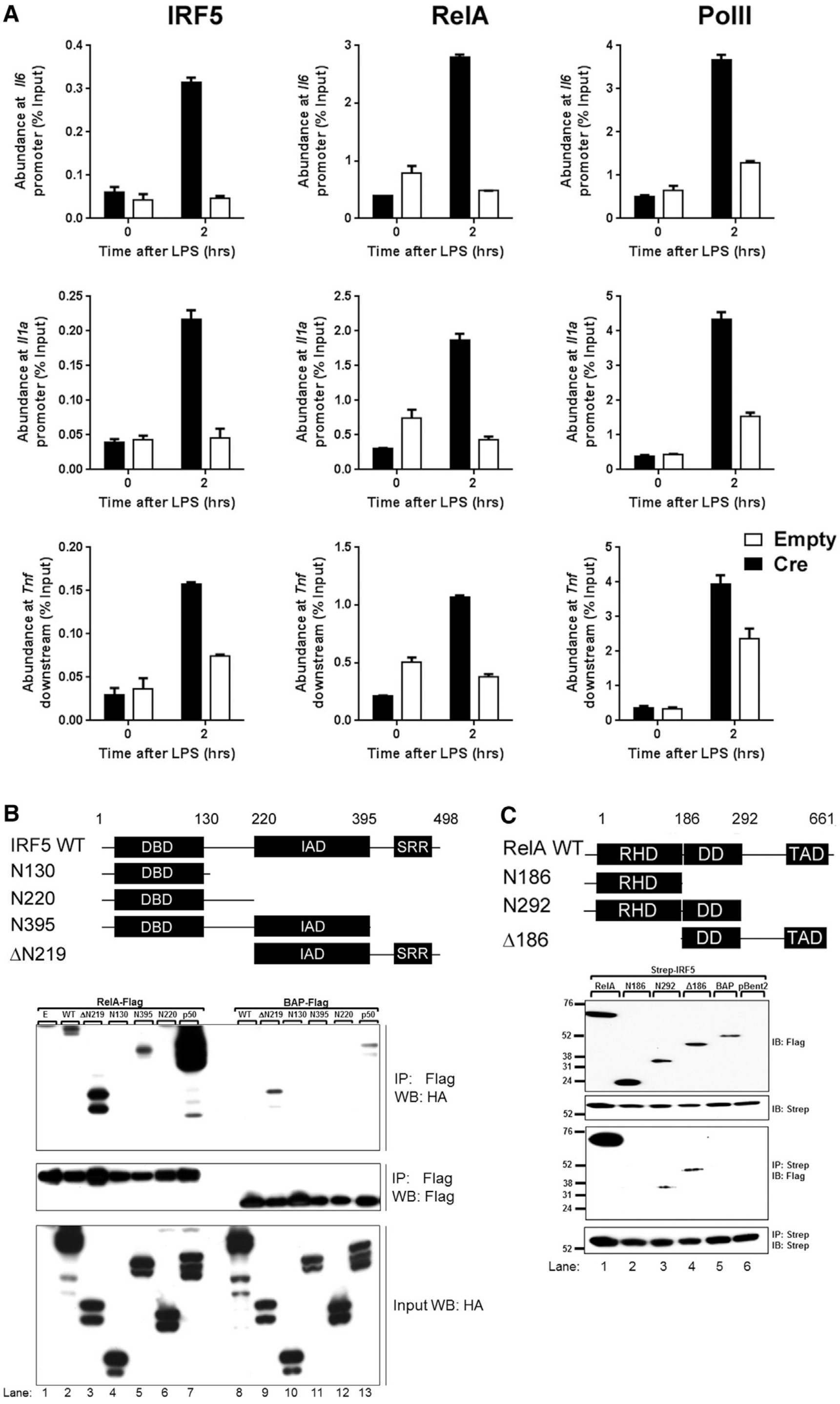

Figure 5. IRF5:RelA Interaction Occurs via the IRF IAD and RelA DD

(A) GM-BMDMs from RelAFl/Fl mice were infected with either Cre or Empty adenovirus and stimulated with LPS (100 ng/ml) for 2 hr or left unstimulated and used in ChIP analysis for IRF5, RELA, or PolII recruitment on *Il-6*, *Il-1a*, and *TNF* loci. Data indicate mean percent input relative to genomic DNA ± SD of a representative experiment.

(B) Schematic of IRF5 truncation mutants prepared by PCR using IRF5 cDNA as a template. Each mutant was cloned into pBent vector with One-STrEP and HA tags at the N terminus. HEK293-TLR4-Md2/CD14 cells were cotransfected with either RelA-Flag (lanes 1–7) or Bap-Flag (lanes 8–13) and each of the IRF5 truncation mutants (lanes 2–6 and 8–12) or p50 (lane 7 and 13) as a positive control. Following immunoprecipitation (IP) on M2 anti-Flag beads, the Flag peptide eluates were immunoblotted for IRF5 truncation mutants (anti-HA antibody; top panel) and bait (anti-Flag antibody; middle panel). Immunoblots of input lysate show equal expression of bait IRF5 truncation mutants (anti-HA; bottom panel). RelA interacts with IRF5 WT (lane 2) and truncation mutants possessing the IAD (lanes 3 and 5), but not mutants lacking the IAD (lanes 4 and 6). WB, western blot.

(C) Schematic of RelA truncation mutants prepared by PCR using IRF5 cDNA as a template. Each mutant was cloned into pBent vector with FLAG tag at the N terminus. HEK293-TLR4-Md2/CD14 cells were cotransfected with full-length RelA-FLAG (lane 1), truncated RelA-FLAG mutants (lanes 2–3), BAP-FLAG (lane 4), or pBent (lane 5) and One-STrEP-IRF5. Immunoblots of input lysate show equal expression of prey RelA truncation mutants (anti-FLAG; bottom panel). Following affinity purification on Strep-Tactin beads, the biotin eluates were immunoblotted for RelA truncation mutants (anti-FLAG; top panel). Immunoblots of the eluates show equal recovery of the bait One-STrEP-IRF5 (middle panel). IRF5 interacts with RelA WT and truncation mutants containing DD (lanes 1, 3, and 4), but not mutants lacking the DD (lane 2).

See also Figure S5.

inflammatory activities, without having deleterious effects on the whole innate immunity that is an essential first line of defense against microbes.

Binding of a pioneer TF, PU.1, in macrophages is thought to be sufficient to promote the deposition of H3K4me1 and to create small open regions of accessible DNA that can be bound by stress-inducible dynamic TFs, such as NF-κB and IRFs (i.e., enhancers) (Natoli et al., 2011). Less clear is whether PU.1 also promotes the deposition of H3K4me3 and chromatin opening at gene promoters. We find that PU.1 binding corresponds to the peaks of both H3K4me1 and H3K4me3 deposition and demonstrates bimodal distribution of histone marks indicative of nucleosome depletion. Binding of IRF5:RelA is significantly enriched on PU.1-marked regulatory elements. This is in line with previous studies that indicated that a subset of macrophage PU.1-marked enhancers was enriched for both NF-κB and IRFs (Ghisletti et al., 2010). Moreover, we find that the IRF5:RelA cistrome encompasses a noncanonical composite PU.1:ISRE. We observed that IRF5 can also

physically interact with PU.1 (data not shown), whereas others recently demonstrated that a preferred mode of IRF5 binding may actually be to a half ISRE site (Jolma et al., 2013). More work is needed to tease out the dynamics of RelA and PU.1 involvement in the binding of IRF5, but it is highly likely that PU.1 binds first because it functions as a pioneering factor in macrophages (Garber et al., 2012; Ghisletti et al., 2010; Heinz et al., 2010), followed by the binding of RelA at inflammatory gene loci and subsequently docking of IRF5. Thus, in inflammatory macrophages, IRF5 imposes yet another previously unobserved level of control on the transcriptional network.

Can this be a mode of binding at other gene loci identified in this study as IRF5 but not RelA targets? The interactome of IRF5 is rapidly expanding (Eames et al., 2012; Feng et al., 2010); thus, it is possible that other yet to be identified TFs may aid to recruit IRF5 to other gene promoters in the absence of RelA binding. We found a strong enrichment in SP1-binding motif under IRF5 ChIP-seq peaks, suggesting that this factor might be involved in high-order transcriptional complexes containing IRF5. Supporting this model is a recent analysis of IRF5 binding in human peripheral blood mononuclear cells stimulated with immune complexes that also identified Sp1 as one of the major motifs in IRF5 target regions (Wang et al., 2013).

It is possible that IRF5 acts similarly to another member of the IRF family, IRF3, which was shown to promote transcription by removing a nucleosome barrier (Ramirez-Carrozzi et al., 2009). IRF5 interacts with a number of chromatin modifiers including acetyltransferases (CBP/300), which were specifically recruited to the interferon α promoter in response to viral induction (Feng et al., 2010). However, it is unlikely that IRF5 plays a causative role in the initial chromatin remodeling because binding of NF-κB, which assists IRF5 in recruitment to inflammatory gene loci, requires nucleosome-free DNA (Lone et al., 2013; Natoli, 2012). Can IRF5 play a role in chromatin remodeling at later stages of gene expression? We have recently demonstrated that IRF5 interacts with KAP1 to indirectly recruit SETDB1 methyltransferase, ultimately leading to deposition of H3K9me3—a mark of transcriptional repression (Eames et al., 2012).

IRF5 is a genetic risk factor for many autoimmune diseases, including systemic lupus erythematosus, rheumatoid arthritis, multiple sclerosis, and inflammatory bowel disease (Dideberg et al., 2007; Dieguez-Gonzalez et al., 2008; Graham et al., 2006; Kristjansdottir et al., 2008). Anti-inflammatory drugs that target molecules that are pivotal to the inflammatory process, like TNF and COX2, have proved successful, but the ultimate aim would be to target transcription of a specific subset of proinflammatory genes (Smale, 2010). Inhibiting IRF5 activity may pave the way for the development of more selective drugs targeting the basic mechanisms underlying the inflammatory response.

EXPERIMENTAL PROCEDURES

Mice

The generation of *Irf5*$^{-/-}$ mice has been described by Takaoka et al. (2005). All procedures were approved by the Ethical Review Process Committee and the UK Home Office, in accordance with the Animals (Scientific Procedures) Act 1986.

Cell Culture

For the generation of M1 macrophages differentiated with GM-CSF, bone marrow of WT C57Bl6 mice was cultured in RPMI-1640 medium (PAA Laboratories) supplemented with recombinant mouse GM-CSF (20 ng/ml; PeproTech). After 8 days, cells were washed with PBS and replated, then stimulated with LPS (100 ng/ml; Alexis Biochemicals).

ChIP-Seq

Nuclear lysates of formaldehyde-fixed GM-CSF macrophages were isolated as described previously by De Santa et al. (2007). Each lysate was immunoprecipitated with 10 μg of the following antibodies: IRF5 (Abcam; ab21689), RelA (Santa Cruz Biotechnology; sc-372), and PolII (Santa Cruz; sc-899). ChIP was performed for each antibody as described previously by Ghisletti et al. (2010).

Please note that independent IRF5 ChIP-seq data sets were recently generated by us in WT and IRF5$^{-/-}$ GM-BMDMs with 50 bp paired-end sequencing following stimulation with LPS for 0 and 2 hr. Preliminary analysis of these data sets by MACS2 algorithm with filtering out of peaks detected in the IRF5$^{-/-}$ from the WT corroborated the findings reported in this manuscript. Further details of next-generation sequencing and analysis are provided in the Supplemental Experimental Procedures.

Microarray Analysis

Microarray (accession number E-MTAB-2032) data were analyzed in R/bioconductor using the beadarray (version 2.4.2; Dunning et al., 2007). Differentially expressed genes were called with SAM method (Tusher et al., 2001) applying an FDR threshold of 10%.

ACCESSION NUMBERS

The ArrayExpress accession numbers for the microarray data, single-end ChIP seq data, and paired-end ChIP-seq data reported in this paper are, respectively, E-MTAB-2032, E-MTAB-2031, and E-MTAB-2661.

SUPPLEMENTAL INFORMATION

Supplemental Information includes Supplemental Experimental Procedures, five figures, and three tables and can be found with this article online at http://dx.doi.org/10.1016/j.celrep.2014.07.034.

AUTHOR CONTRIBUTIONS

D.G.S. designed research, performed the wet lab experiments, and wrote the paper. A.H. performed bioinformatics analysis of the data and helped write and edit the manuscript.

ACKNOWLEDGMENTS

This work was supported by the MRC project grant (MR/J001899/1) (to D.G.S. and I.A.U.) and The Kennedy Institute Trustees Research Fund (to H.L.E., M.W., and K.B.). A.H. was funded by the MRC CGAT Programme (G1000902). We would like to thank Professors Chris Ponting, Steve Smale, and Gioacchino Natoli for critical comments on the manuscript and helpful suggestions.

Received: September 13, 2013
Revised: January 10, 2014
Accepted: July 22, 2014
Published: August 21, 2014

REFERENCES

Badis, G., Berger, M.F., Philippakis, A.A., Talukder, S., Gehrke, A.R., Jaeger, S.A., Chan, E.T., Metzler, G., Vedenko, A., Chen, X., et al. (2009). Diversity and complexity in DNA recognition by transcription factors. Science *324*, 1720–1723.

Brass, A.L., Kehrli, E., Eisenbeis, C.F., Storb, U., and Singh, H. (1996). Pip, a lymphoid-restricted IRF, contains a regulatory domain that is important for autoinhibition and ternary complex formation with the Ets factor PU.1. Genes Dev. *10*, 2335–2347.

De Santa, F., Totaro, M.G., Prosperini, E., Notarbartolo, S., Testa, G., and Natoli, G. (2007). The histone H3 lysine-27 demethylase Jmjd3 links inflammation to inhibition of polycomb-mediated gene silencing. Cell *130*, 1083–1094.

Dideberg, V., Kristjansdottir, G., Milani, L., Libioulle, C., Sigurdsson, S., Louis, E., Wiman, A.C., Vermeire, S., Rutgeerts, P., Belaiche, J., et al. (2007). An insertion-deletion polymorphism in the interferon regulatory Factor 5 (IRF5) gene confers risk of inflammatory bowel diseases. Hum. Mol. Genet. *16*, 3008–3016.

Dieguez-Gonzalez, R., Calaza, M., Perez-Pampin, E., de la Serna, A.R., Fernandez-Gutierrez, B., Castañeda, S., Largo, R., Joven, B., Narvaez, J., Navarro, F., et al. (2008). Association of interferon regulatory factor 5 haplotypes, similar to that found in systemic lupus erythematosus, in a large subgroup of patients with rheumatoid arthritis. Arthritis Rheum. *58*, 1264–1274.

Dunning, M.J., Smith, M.L., Ritchie, M.E., and Tavare, S. (2007). beadarray: R classes and methods for Illumina bead-based data. Bioinformatics *23*, 2183–2184.

Eames, H.L., Saliba, D.G., Krausgruber, T., Lanfrancotti, A., Ryzhakov, G., and Udalova, I.A. (2012). KAP1/TRIM28: an inhibitor of IRF5 function in inflammatory macrophages. Immunobiology *217*, 1315–1324.

Escalante, C.R., Brass, A.L., Pongubala, J.M., Shatova, E., Shen, L., Singh, H., and Aggarwal, A.K. (2002). Crystal structure of PU.1/IRF-4/DNA ternary complex. Mol. Cell *10*, 1097–1105.

Feng, D., Sangster-Guity, N., Stone, R., Korczeniewska, J., Mancl, M.E., Fitzgerald-Bocarsly, P., and Barnes, B.J. (2010). Differential requirement of histone acetylase and deacetylase activities for IRF5-mediated proinflammatory cytokine expression. J. Immunol. *185*, 6003–6012.

Fleetwood, A.J., Lawrence, T., Hamilton, J.A., and Cook, A.D. (2007). Granulocyte-macrophage colony-stimulating factor (CSF) and macrophage CSF-dependent macrophage phenotypes display differences in cytokine profiles and transcription factor activities: implications for CSF blockade in inflammation. J. Immunol. *178*, 5245–5252.

Garber, M., Yosef, N., Goren, A., Raychowdhury, R., Thielke, A., Guttman, M., Robinson, J., Minie, B., Chevrier, N., Itzhaki, Z., et al. (2012). A high-throughput chromatin immunoprecipitation approach reveals principles of dynamic gene regulation in mammals. Mol. Cell *47*, 810–822.

Ghisletti, S., Barozzi, I., Mietton, F., Polletti, S., De Santa, F., Venturini, E., Gregory, L., Lonie, L., Chew, A., Wei, C.L., et al. (2010). Identification and characterization of enhancers controlling the inflammatory gene expression program in macrophages. Immunity *32*, 317–328.

Gordân, R., Hartemink, A.J., and Bulyk, M.L. (2009). Distinguishing direct versus indirect transcription factor-DNA interactions. Genome Res. *19*, 2090–2100.

Graham, R.R., Kozyrev, S.V., Baechler, E.C., Reddy, M.V., Plenge, R.M., Bauer, J.W., Ortmann, W.A., Koeuth, T., González Escribano, M.F., Pons-Estel, B., et al.; Argentine and Spanish Collaborative Groups (2006). A common haplotype of interferon regulatory factor 5 (IRF5) regulates splicing and expression and is associated with increased risk of systemic lupus erythematosus. Nat. Genet. *38*, 550–555.

Heinz, S., Benner, C., Spann, N., Bertolino, E., Lin, Y.C., Laslo, P., Cheng, J.X., Murre, C., Singh, H., and Glass, C.K. (2010). Simple combinations of lineage-determining transcription factors prime cis-regulatory elements required for macrophage and B cell identities. Mol. Cell *38*, 576–589.

Jolma, A., Kivioja, T., Toivonen, J., Cheng, L., Wei, G., Enge, M., Taipale, M., Vaquerizas, J.M., Yan, J., Sillanpää, M.J., et al. (2010). Multiplexed massively parallel SELEX for characterization of human transcription factor binding specificities. Genome Res. *20*, 861–873.

Jolma, A., Yan, J., Whitington, T., Toivonen, J., Nitta, K.R., Rastas, P., Morgunova, E., Enge, M., Taipale, M., Wei, G., et al. (2013). DNA-binding specificities of human transcription factors. Cell *152*, 327–339.

Krausgruber, T., Saliba, D., Ryzhakov, G., Lanfrancotti, A., Blazek, K., and Udalova, I.A. (2010). IRF5 is required for late-phase TNF secretion by human dendritic cells. Blood *115*, 4421–4430.

Krausgruber, T., Blazek, K., Smallie, T., Alzabin, S., Lockstone, H., Sahgal, N., Hussell, T., Feldmann, M., and Udalova, I.A. (2011). IRF5 promotes inflammatory macrophage polarization and TH1-TH17 responses. Nat. Immunol. *12*, 231–238.

Kristjansdottir, G., Sandling, J.K., Bonetti, A., Roos, I.M., Milani, L., Wang, C., Gustafsdottir, S.M., Sigurdsson, S., Lundmark, A., Tienari, P.J., et al. (2008). Interferon regulatory factor 5 (IRF5) gene variants are associated with multiple sclerosis in three distinct populations. J. Med. Genet. *45*, 362–369.

Lim, C.A., Yao, F., Wong, J.J., George, J., Xu, H., Chiu, K.P., Sung, W.K., Lipovich, L., Vega, V.B., Chen, J., et al. (2007). Genome-wide mapping of RELA(p65) binding identifies E2F1 as a transcriptional activator recruited by NF-kappaB upon TLR4 activation. Mol. Cell *27*, 622–635.

Lone, I.N., Shukla, M.S., Charles Richard, J.L., Peshev, Z.Y., Dimitrov, S., and Angelov, D. (2013). Binding of NF-κB to nucleosomes: effect of translational positioning, nucleosome remodeling and linker histone H1. PLoS Genet. *9*, e1003830.

Luedde, T., Heinrichsdorff, J., de Lorenzi, R., De Vos, R., Roskams, T., and Pasparakis, M. (2008). IKK1 and IKK2 cooperate to maintain bile duct integrity in the liver. Proc. Natl. Acad. Sci. USA *105*, 9733–9738.

Machanick, P., and Bailey, T.L. (2011). MEME-ChIP: motif analysis of large DNA datasets. Bioinformatics *27*, 1696–1697.

Mangan, S., and Alon, U. (2003). Structure and function of the feed-forward loop network motif. Proc. Natl. Acad. Sci. USA *100*, 11980–11985.

Natoli, G. (2012). NF-κB and chromatin: ten years on the path from basic mechanisms to candidate drugs. Immunol. Rev. *246*, 183–192.

Natoli, G., Ghisletti, S., and Barozzi, I. (2011). The genomic landscapes of inflammation. Genes Dev. *25*, 101–106.

Ostuni, R., and Natoli, G. (2011). Transcriptional control of macrophage diversity and specialization. Eur. J. Immunol. *41*, 2486–2490.

Paun, A., Bankoti, R., Joshi, T., Pitha, P.M., and Stäger, S. (2011). Critical role of IRF-5 in the development of T helper 1 responses to *Leishmania donovani* infection. PLoS Pathog. *7*, e1001246.

Ponjavic, J., Oliver, P.L., Lunter, G., and Ponting, C.P. (2009). Genomic and transcriptional co-localization of protein-coding and long non-coding RNA pairs in the developing brain. PLoS Genet. *5*, e1000617.

Ramirez-Carrozzi, V.R., Braas, D., Bhatt, D.M., Cheng, C.S., Hong, C., Doty, K.R., Black, J.C., Hoffmann, A., Carey, M., and Smale, S.T. (2009). A unifying model for the selective regulation of inducible transcription by CpG islands and nucleosome remodeling. Cell *138*, 114–128.

Rashid, N.U., Giresi, P.G., Ibrahim, J.G., Sun, W., and Lieb, J.D. (2011). ZINBA integrates local covariates with DNA-seq data to identify broad and narrow regions of enrichment, even within amplified genomic regions. Genome Biol. *12*, R67.

Siggers, T., Chang, A.B., Teixeira, A., Wong, D., Williams, K.J., Ahmed, B., Ragoussis, J., Udalova, I.A., Smale, S.T., and Bulyk, M.L. (2012). Principles of dimer-specific gene regulation revealed by a comprehensive characterization of NF-κB family DNA binding. Nat. Immunol. *13*, 95–102.

Smale, S.T. (2010). Selective transcription in response to an inflammatory stimulus. Cell *140*, 833–844.

Takaoka, A., Yanai, H., Kondo, S., Duncan, G., Negishi, H., Mizutani, T., Kano, S., Honda, K., Ohba, Y., Mak, T.W., and Taniguchi, T. (2005). Integral role of IRF-5 in the gene induction programme activated by Toll-like receptors. Nature *434*, 243–249.

Tamura, T., Thotakura, P., Tanaka, T.S., Ko, M.S., and Ozato, K. (2005). Identification of target genes and a unique cis element regulated by IRF-8 in developing macrophages. Blood *106*, 1938–1947.

Tamura, T., Yanai, H., Savitsky, D., and Taniguchi, T. (2008). The IRF family transcription factors in immunity and oncogenesis. Annu. Rev. Immunol. *26*, 535–584.

Tusher, V.G., Tibshirani, R., and Chu, G. (2001). Significance analysis of microarrays applied to the ionizing radiation response. Proc Natl Acad Sci USA *98*, 5116–5121.

Wang, C., Sandling, J.K., Hagberg, N., Berggren, O., Sigurdsson, S., Karlberg, O., Rönnblom, L., Eloranta, M.L., and Syvänen, A.C. (2013). Genome-wide profiling of target genes for the systemic lupus erythematosus-associated transcription factors IRF5 and STAT4. Ann. Rheum. Dis. *72*, 96–103.

Weiss, M., Blazek, K., Byrne, A.J., Perocheau, D.P., and Udalova, I.A. (2013). IRF5 is a specific marker of inflammatory macrophages in vivo. Mediators Inflamm. *2013*, 245804.

Wong, D., Teixeira, A., Oikonomopoulos, S., Humburg, P., Lone, I.N., Saliba, D., Siggers, T., Bulyk, M., Angelov, D., Dimitrov, S., et al. (2011). Extensive characterization of NF-κB binding uncovers non-canonical motifs and advances the interpretation of genetic functional traits. Genome Biol. *12*, R70.

Supplemental Experimental Procedures

Plasmids

Expression construct encoding full-length IRF5 with HA and Onestrep-tags were described in Krausgruber et al (2010). The plasmids encoding IRF5 N220, IRF5ΔDBD and IRF5ΔN219 are described in Eames et al., 2012. HA-tagged deletion mutants - IRF5 N130 (aa 1-130) and N395 (aa 1-395) - were amplified by PCR from the full-length human IRF5 cDNA and inserted into pBent2-strep vector. Flag-tagged deletion mutants – RelA N186 (aa1-186), RelA N292 (aa1-292) and RelA Δ186 (aa186-551) – were amplified by PCR from plasmid DNA encoding full-length RelA-Flag (described in Krausgruber et al., 2010), and inserted into the pBent2 vector. All constructs were verified by DNA sequencing. The sequences and restriction maps are available upon request.

Cell Culture

For the generation of M1 macrophages differentiated with GM-CSF, bone marrow of wild type C57Bl6 mice was cultured in RPMI-1640 medium (PAA Laboratories) supplemented with recombinant mouse GM-CSF (20 ng/ml; Preprotech). After 8 d, cells were washed with PBS and replated, then stimulated with LPS (100 ng/ml; Alexis Biochemicals).

Adenoviral Cre-mediated Rela knockdown

For the Cre-mediated conditional knockdown of RelA, M1 macrophages from RelAFl/Fl mice were differentiated as described above. After 7 days cells were washed with PBS and replated in 10 cm plates (10^7 cells per plate) in antibiotics-free RPMI containing either Cre or empty vector (pBent2) adenoviral particles at a multiplicity of infection (MOI) of 50:1 in a final volume of 5 mL. The plates were incubated for 4hrs at 37°C followed by the addition of 5mL standard media per plate. Cells were allowed to recover for a further 12 hrs before being left unstimulated or stimulated with LPS (100 ng/ml; Alexis Biochemicals).

ChIP-seq

50, 100 and 300 million GM-CSF derived M1 macrophages were used for each Pol II, RelA and IRF5 ChIP experiment, respectively. Cells were fixed for 10

minutes with 1 % formaldehyde, quenched with 125 mM of Tris pH 7.5 and washed with ice-cold PBS. Nuclear lysates were isolated as described previously (De Santa et al. 2007) and sonicated with a Bioruptor (Diagenode) to obtain chromatin fragment sizes that average 500bp. Each lysate was immunoprecipitated with 10 µg of the following antibodies: IRF5 (Abcam; ab21689), RelA (Santa Cruz; sc-372) and PolII (Santa Cruz; sc-899). ChIP was performed for each antibody as described previously (Ghisletti et al. 2010). ChIPped DNA was quantified with the Quant-iT dsDNA High Sensitivity Assay Kit (Invitrogen #Q33120). DNA yields ranged from 10 – 20 ng. The ChIP-Seq datasets were generated using 33bp single end sequencing (Accession number: E-MTAB-2033). **NOTE:** independent IRF5 ChIP-seq datasets were recently generated by us in WT and IRF5-/- GM-BMDMs with 50bp paired end sequencing following stimulation with LPS for 0 and 2hrs in duplicate. 13-24 (mean = 22) million reads were mapped to the genome for each experimental condition. (Accession number: E-MTAB-2661). Using MACS2 algorithm at 20% FDR we detected 417 and 533 peaks in duplicates of IRF5+/+ datasets, and 461 and 457 peaks in duplicates of IRF5-/- datasets at 0h. Following 2h of LPS stimulation 1453 and 2345 peaks were detected in duplicates of IRF5+/+ datasets, while the number of peaks in IRF5-/- datasets remained largely unchanged (319 and 372 peaks). Combining IRF5+/+ datasets at 2 h post stimulation amounted to 2835 peaks, while combining IRF5-/- dataset resulted in 497 peaks. Thus, ~15% of IRF5 ChIP-Seq peaks were false positive, with remaining 2538 IRF5 binding peaks being bona fide peaks. Preliminary cross-validation analyses using this dataset corroborated the findings reported in this manuscript.

ChIP-qPCR of Cre-mediated RelA Knockdown

A total of $1x10^7$ Cre or Empty adenovirus infected cells described above were used for ChIP-qPCR. Sonicated nuclear lysates were prepared as in ChIP-seq procedure described above. Each lysate was immunoprecipitated with 3 µg of either Pol II, or IRF5 or RelA. The immunoprecipitated DNA fragments were then interrogated by real-time PCR using SYBR Premix Ex Taq II master mix (Takara Bio) and the following primers: Il1a (TGCCCAGTCTGTCCCTCCTCATGCT and CCCGAGCTTTGGCTCCAGTCTGCT); Il1b (GGATGTGCGGAACAAAGGTAGGCACG and ACTCCAACTGCAAAGCTCCCTCAGC) and TNF.

Data were analyzed using ABI 7900HT machine (Applied Biosystems, USA). All primer sets were tested for specificity and equal efficiency before use.

Coimmunoprecipitation

(i) *Identifcation IRF5 domain that interacts with RelA*: HEK-293–TLR4-CD14/Md2 cells were co-transfected with ONEstrep tagged deletion IRF5 mutant or WT constructs and either FLAG tagged RelA or control Bacterial Alkaline Phosphatase (BAP). 24 hours post-transfection cells cells were lysed in RIPA buffer and immunoprecipitated on anti-FLAG M2 sepharose beads (Sigma). Flag peptide eluates were immunoblotted with anti-FLAG-HRP (A8952; Sigma) for bait or anti-HA-HRP (12013819001; Roche) for prey IRF5 proteins.

(ii) *Identification of RelA domain that interacts with IRF5*: HEK-293–TLR4-CD14/Md2 cells were co-transfected with ONEstrep tagged IRF5 WT construct and FLAG tagged deletion RelA mutants. 24 hours post-transfection cells were lysed in 1% TX-100 lysis buffer (containing) and affinity purified on Strep-Tactin Macrprep sepharose (IBA). Biotin eluates were immunoblotted with anti-Strep (IBA) for bait IRF5 and anti-FLAG –HRP (A8952; Sigma) for prey RelA proteins.

Protein Binding Microarrays (PBMs)

Sequences of the different primers and DNA ligands can be found in Additional file "primers_oligos_IRF". All quantification of nucleic acid samples was performed according to manufacturer instructions on a Qubit Fluorometer (Invitrogen #Q32857, Paisley, United Kingdom) and with either the Quant-iT dsDNA High Sensitivity Assay Kit (Invitrogen #Q33120) or the Quant-iT dsDNA Broad Range Assay Kit (Invitrogen #Q33130). Protein assays were performed using the Quant-iT™ Protein Assay Kit (Invitrogen #Q33210).

Protein expression and purification

Expression constructs for the IRF proteins (*Homo sapiens*) used in this study were created following a set of procedures previously established by Udalova and co-workers [42]. Briefly, pET vectors for expression in BL21 (DE3) *Escherichia coli* (Merck, Nottingham, United Kingdom) were used to produce

histidine-tagged (His-tagged) recombinant proteins. Proteins were over-expressed through induction with 0.2 mM isopropyl β-D-1-thiogalactopyranoside (IPTG) at 30°C for 5 hours. Pellets of cells were harvested in 'Ni-NTA binding' buffer with added EDTA-free protease inhibitor (Roche, West Sussex, United Kingdom), pulse-sonicated for 2 minutes and debris removed via centrifugation at 16,000 *g*. A two-step purification procedure was then employed, first with the 'Ni-NTA His-Bind Resin' system (Merck #70666) and then a subsequent purification based on DNA-affinity isolation of functional, DNA-binding protein. Ni-NTA purification was carried out according to the manufacturer's guidelines. For DNA-affinity isolation, the processing of a sample derived from 250 ml of bacteria culture required 0.128 μM of oligonucleotides specific for IRF protein binding. Prior to use, the oligonucleotides were annealed via incubation in NEB Buffer 3 at 94°C for 1 minute then subsequently for an additional 69 cycles of 1 minute each coupled to a per-cycle, step-wise decrease of 1°C. A pre-annealed oligo mixture (712.5 μl) was conjugated with streptavidin-agarose (Sigma, Dorset, United Kingdom) before once-purified material from the preceding step was added to it.

ChIP-Seq analysis

Reads were mapped onto mouse genome build 37 by NCBI and the Mouse Genome Consortium (Church et al., 2009) , downloaded from UCSC (Fujita et al., 2011), mm9) using bowtie 0.12.7 (Langmead et al., 2009)) with the following options:-n 2 -a --best --strata -m 1. Peaks were called with Zinba (version 2.02.01, (Rashid et al., 2011)) using default options, a window size of 200 and an FDR of 1%. Aligned reads and called peaks were visualized with IGV (Thorvaldsdottir et al., 2013). After visualization we noticed that abundant peaks in the 3' region of genes caused binding events in the 5' region of genes to go undetected. For the promoter analysis only we thus called peaks using MACS2 in a region of -10kb, 1kb around transcription start sites only. This added an additional 939 peaks in the Irf5 data set. Read densities were analyzed with in-house scripts.

Microarray analysis

Microarray (Accession number: E-MTAB-2032)data was analysed in R/bioconductor using the beadarray (version 2.4.2, Ref (Dunning et al., 2007))

and siggenes packages (version 1.28.0, Schwender 2012). Differentially expressed genes were called with SAM method (Ref (Tusher et al., 2001)) applying a false discovery rate threshold of 10%.

Interaction analysis and genomic enrichment

The significance of genomic enrichment was analysed using a simulation procedure similar to (Ponjavic et al., 2009). Briefly, the genomic association between a test set of peaks and a genomic annotation is measured by randomly simulating sets of peaks of equivalent size and length distribution to the test set. Enrichment and depletion are measured as ratio of the observed nucleotide overlap compared to the expected nucleotide overlap from 10,000 simulated sets and its significance is expressed as a P-Value. The significance of fold change difference is computed in an analogous manner by combining the results from two parallel simulations. Genomic regions of low mapability are excluded from the simulation. To control for biases in gene density, the overlap with chromatin marks was assessed in 50kb regions around genes only. For the overlap with transcription factors only regions 2kb upstream and 0.5kb downstream of transcription start sites were considered. The code for the simulations is publicly available (http://code.google.com/p/genomic-association-tester/).

Motif analysis

Motif analysis in ChIP-Seq peaks was performed using MEME-ChIP(Machanick and Bailey, 2011). Motif discovery was performed on the top 500 peaks using MEME-ChIP in 200 bp windows around the position with highest read density in a ChIP-Seq peak. Motif discovery used both repeat masked and unmasked sequence using the following options: "-dna -revcomp -mod anr -nmotifs 3 -minw 5 -maxw 30".

Protein binding microarrays

We designed 2 × 105K Agilent arrays using eArray (details given below). These arrays were comprised of two main sets of probes: 12-mer sequences designed for IRF binding and a set of 11-mer sequences design for NF-kB binding use for validation purposes. As an IRF consensus sequence, we used the motif NRWANNGARAVY that codes for a total of 3072 different motifs.

Experiments were carried out in technical replicates showing a 98% correlation. Z-scores were assigned to each sequence represented in the array. The sequences were ranked and used to produce binding motifs over all 12bp using weblogo (http://weblogo.berkeley.edu/)

Microarrays (PBMs)
Description of probe-design on the microarrays.
Our microarrays are chips of 2 arrays each with 104961 probes per array. Each array contains 1325 manufacturer-probes (Agilent) and 103636 customized probes. Each probe is represented using 4 different flanks of 4-nt length: AGCT, ATGA, AGTC, AGAT and each flanked probe is replicated 7 times. Additional "IRF_design_microarray.txt" shows a breakdown of the number and type of probes present on each array.

Protocol for generation and use of double-stranded protein microarrays.
Single stranded probes on each array were rendered double-stranded with the following procedure. For each array on a 2x150K chip, 820 µl of "ds-mix" (NEB buffer 2, 0.1 µM dsPrimer, 2.5 X BSA, 163 µM dNTPs, 1.63 µM of Cy3-dCTP and 27.2 U of Klenow DNA polymerase I) was dispensed onto a "1x205K gasket", combined with a chip, the entire unit sealed within a hybridization chamber and incubated within a rotating-oven at 37 ºC for 90 min. The following washes were then carried out: 6 washes in 0.01 % Triton-X/PBS for 3 min each followed by a 3 min wash in PBS. Arrays were dried via centrifugation. To ascertain overall success of the procedure, arrays were scanned using the Agilent Microarray Scanner at maximum power and the image analyzed for extent of Cy3-incorporation within individual probes.
Prior to hybridisation, arrays were blocked, washed according to manufacturer's guidelines and incubated in 2 % milk/PBS for 1 h at room temperature. This was followed by 2 washes (6 min each; 0.1% Tween-20/PBS followed by 0.01% Triton X-100/PBS) and ended with a brief rinse in water before drying via centrifugation. Hybridizations were performed using a protein concentration of 0.01 µg/µl in 420 µl of protein binding reaction mix (10 mM HEPES pH 8, 0.5 M NH_4OAC, 100 mM NaCl, 5 mM $MgCl_2$/MgAcetate, 1 mM DTT and 5% glycerol). Protein binding reaction mixes were dispensed into the different compartments of a 2x105K gasket slide (Agilent), combined with a chip and the entire unit sealed into a hybridization chamber. The assembled

unit was rotated in the hybridization oven for 1 h at room temperature. Arrays were then subsequently washed 6 times with 1 % Tween-20/PBS for 6 min each and a further 6 washes with 0.01 % Triton X-100/PBS for 6 min each. This was followed by a brief rinse in water and drying via centrifugation. Labelling of bound protein was carried out in two stages. Firstly, arrays were incubated with 0.8 µg of primary rabbit anti-His antibody (Santa Cruz) in a 2 % milk/PBS solution for 1 h at room temperature. This was followed by 6 washes with 0.05 % Tween-20/PBS for 3 min each and other 6 washes with 0.01 % Triton X-100/PBS for 3 min. Subsequently, arrays were incubated with 6 µg of secondary Cy5-conjugated anti-rabbit IgG antibody in a 2 % milk/PBS solution for 30 min at 37 °C before being washed as per above. Before drying, arrays were first rinsed in PBS for 6 mins and then briefly again in water.
Arrays were dried via centrifugation and scanned using the Agilent Microarray Scanner at maximum power.

Genotyping

The IRF5$^{-/-}$ line was genotyped for the DOCK2 mutation as described previously (Yasuda et al., 2013). Briefly, DNA was obtained from ear clips using REDExtract-N-Amp (Sigma) and PCR was performed using the following primers which detect the DOCK2 mutation as a 305-bp product: DOCK2In29.4F GAC CTT ATG AGG TGG AAC CAC AAC C; DOCK2InR22.3.1R GAT CCA AAG ATT CCC TAC AGC TCC AC. IRF5 mice possessing the mutation for DOCK2 were culled and all experiments were performed on a line that was wild-type for DOCK2.

Accession numbers:

Unprocessed data have been deposited at ArrayExpress under accession numbers E-MTAB-2031 (ChIP-Seq data), E-MTAB-2661 (ChIP-Seq data) and E-MTAB-2032 (microarray data)

SUPPLEMENTAL FIGURES

Figure S1:

(A) Cell surface receptor and cytokine expression in macrophages BMDMs differentiated with GM-CSF or M-CSF. FACS samples were collected at day 9 of differentiation and stained for F4/80, CD206 or MHCII, IRF5 after the cells were stimulated with LPS (100ng/mL; 4hrs) or left unstimulated. Data are representative of 6 experiments. **(B)** Scatter plots of RelA ChIP-seq peaks following LPS stimulation at 0.5 or 2hr with unstimulated condition. **(C)** Scatter plots of IRF5 ChIP-seq peaks following LPS stimulation at 0.5 or 2hr with unstimulated condition. **(D)** Specific Recruitment of IRF5 to example gene promoters (*Il6, ccl5, il12b, il1a, tnf, nfkbia, gadd45b, irf1, il12a* and *mllt6*) were analysed by qPCR in GM-BMDMs from either WT or IRF5 KO mice following LPS stimulation (100 ng/mL; 2hrs). No recruitment of IRF5 was observed on the negative control *Hbb* promoter. Data show mean percentage input relative to genomic DNA (gDNA) plus or minus SD of a representative experiment. **(E)** Average ChIP-seq enrichment profile of IRF5, RelA and Pol II binding regions around transcription start (TSS) and end site (TES) (dotted lines). IRF5, RelA and PolII show distinct binding to upstream of the TSS. IRF5 also displays binding downstream of the TES. **(F)** Binding of RelA, PolII and IRF5 in the 5' or 3' upstream regions of genes. The areas show the proportion of genes that have upstream binding only, downstream binding only, or both. Upstream regions are defined as 5kb upstream from the transcription start site. The downstream regions are similarly defined as 5kb downstream of the transcription termination site.

Figure S2:

(A) Percentage overlap of Pol II intervals with TSS of ENSEMBL gene set were categorised according to absence or presence of IRF5 and RelA peaks. 67% of PolII intervals that contain both a predicted IRF5 and RelA binding event overlap a TSS, while only 32% of PolII intervals without IRF5 and RelA binding overlap a TSS. **(B)** RelA and IRF5 alignment with PU.1 in promoters and enhancers: Intervals were classified according to chromatin marks H3K4Me3 and H3K4Me1 [14] into promoter-like (left panel) and enhancer-like (right panel) groups when the average density of H3K4Me3 > H3KMe1 and H3K4Me1 > H3K4Me3 respectively. Aggregate plots (normalized by total-max & counts) of H3K4Me1, H3K4Me3 and PU.1 (Garber et al., 2012) are centered on the RelA

peaks (Top plot: with IRF5; Middle plot without IRF5) and IRF5 peaks (Bottom plot: without RelA). FDR =1%.

Figure S3:

(A) PCR to detect the DOCK2 mutation. Genomic DNA from $IRF5^{-/-}$ (Left panel, Lanes 1-3) and $IRF5^{+/+}$ (Right panel, Lanes 7-9) did not result in a PCR product for the DOCK2 mutation (305bp; +ve controls Lanes 6 and 10). IRF5 was used as an internal control to verify the adequacy of DNA preparation in each sample (PCR products $IRF5^{-/-}$: 550 bp Lanes 1-3; $IRF5^{+/+}$: 650bp Lanes 7-9; $IRF5^{+/-}$: both 650 and 550 bp).

(B) Gene expression heatmaps of Category 1, 2 and 3 genes affected by IRF5 and RelA KO following LPS stimulation. GM-BMDMs from conventional IRF5 KO (left panel) or conditional RelA KO were each compared to WT controls following stimulation by LPS (100ng/mL) for 0,1,2,4 or 8 hrs; (Data are pooled from three experiments; blue to red represents increase level of gene expression).

Figure S4:

Identification of Consensus IRF Binding Motifs by custom IRF PBMs: Representative DNA-binding site motifs were determined for IRF5 using the total, top 500, top 275 and top 50 motifs bound. Data are derived from pooled data of two experiments.

Figure S5:

q-PCR analysis of IRF5-dependent genes and efficiency RelA knockdown by Cre virus: **(A)** GM-CSF BMDMs from IRF5-/- or WT mice were left stimulated or stimulated with LPS (100ng/mL) for 1,2,4,6 and 8h. *Il1a*, *Il6* and *Tnf* mRNA expression was compared to unstimulated WT control cells. Data shown are the mean ± SD of 4 independent experiments. **(B)** GM-CSF differentiated BMDMs from RelAFL mice were infected with adenoviral vectors encoding Cre or control (Empty) prior to stimulation with LPS (100ng/mL; 2hrs). Approximately 70% of RelA protein was degraded following Cre infection compared to empty control as analysed by Western blotting of RelA. IRF5 protein stability was unaffected following Cre infection

SUPPLEMENTARY TABLES

Table S1:

MappingChIP-seq reads to genome: **(A)** A total of 6 samples were analysed for ChIP-seq following LPS stimulation (100ng/mL) as indicated. For each sample the total number of reads sequenced (total) and the total reads mapped (mapped) are shown. Reads mapping to the exact same position (duplicates) were removed before peak calling. **(B)** The genome was segmented into annotated regions (cds, utr, upstream, downstream, intronic, intergenic) based on the ENSEMBL gene set. To avoid over-counting, an interval is associated with an annotation depending on the location of the peak (the point with the highest read density within an interval) **(C)** and **(D)** To assess whether IRF5 **(C)** and RelA **(D)** intervals are significantly associated with functional genome annotations (described in Figure1b), a simulation procedure was applied (*see Methods*). *Observed* (Observed nucleotide overlap between IRF5/RelA intervals and a genomic region); *Expected* (Expected nucleotide overlap between IRF5/RelA intervals and a genomic region based on simulations); *CI25low/CI95high* (95% confidence intervals); *Stddev* (Standard deviation of expected overlap); *Fold* (Fold change: Observed/Expected); *l2fold* (log2 fold change).

Table S2:

(**A**) Differentially expressed genes are called at FDR= 1% and having a greater than two-fold change in expression following LPS stimulation. **(B)** Fold enrichments of IRF5 and RelA ChIP-seq peaks at chromatin marked regions obtained by simulation procedure (*see Methods*) were used to assess whether IRF5 or RelA were associated with chromatin marks for enhancers (H3K4ME1) or promoters (H3K4ME3) in BMDMs (Barish et al., 2010) and BMDCs (Garber et al., 2012). All enrichments are statistically significant ($p<10^{-4}$) **(C)** Fold enrichments obtained by simulation procedure (*see Methods*) were used to assess degree of overlap of the IRF5:RelA cistrome with PU.1 or PU.1-less marked promoters or enhancers as indicated. All enrichments are statistically significant ($p<10^{-4}$).

Table S3:

Genes affected by conventional IRF5 KO **(A)** and conditional RelA KO **(B)** relative to WT following LPS stimulation split into categories as indicated in Figure 3A. GM-BMDMs from conventional IRF5 KO (left panel) or conditional RelA KO were each compared to WT controls following stimulation by LPS

(100ng/mL) for 0,1,2,4 or 8 hrs; (Data are pooled from three experiments; Down – expression decreased in KO. Up – expression increased in KO; bolding indicates genes affected in both IRF5 and RelA KO).

Barish, G.D., Yu, R.T., Karunasiri, M., Ocampo, C.B., Dixon, J., Benner, C., Dent, A.L., Tangirala, R.K., and Evans, R.M. (2010). Bcl-6 and NF-kappaB cistromes mediate opposing regulation of the innate immune response. Genes Dev *24*, 2760-2765.

Garber, M., Yosef, N., Goren, A., Raychowdhury, R., Thielke, A., Guttman, M., Robinson, J., Minie, B., Chevrier, N., Itzhaki, Z., *et al.* (2012). A high-throughput chromatin immunoprecipitation approach reveals principles of dynamic gene regulation in mammals. Mol Cell *47*, 810-822.

Yasuda, K., Nundel, K., Watkins, A.A., Dhawan, T., Bonegio, R.G., Ubellacker, J.M., Marshak-Rothstein, A., and Rifkin, I.R. (2013). Phenotype and function of B cells and dendritic cells from interferon regulatory factor 5-deficient mice with and without a mutation in DOCK2. International immunology *25*, 295-306.

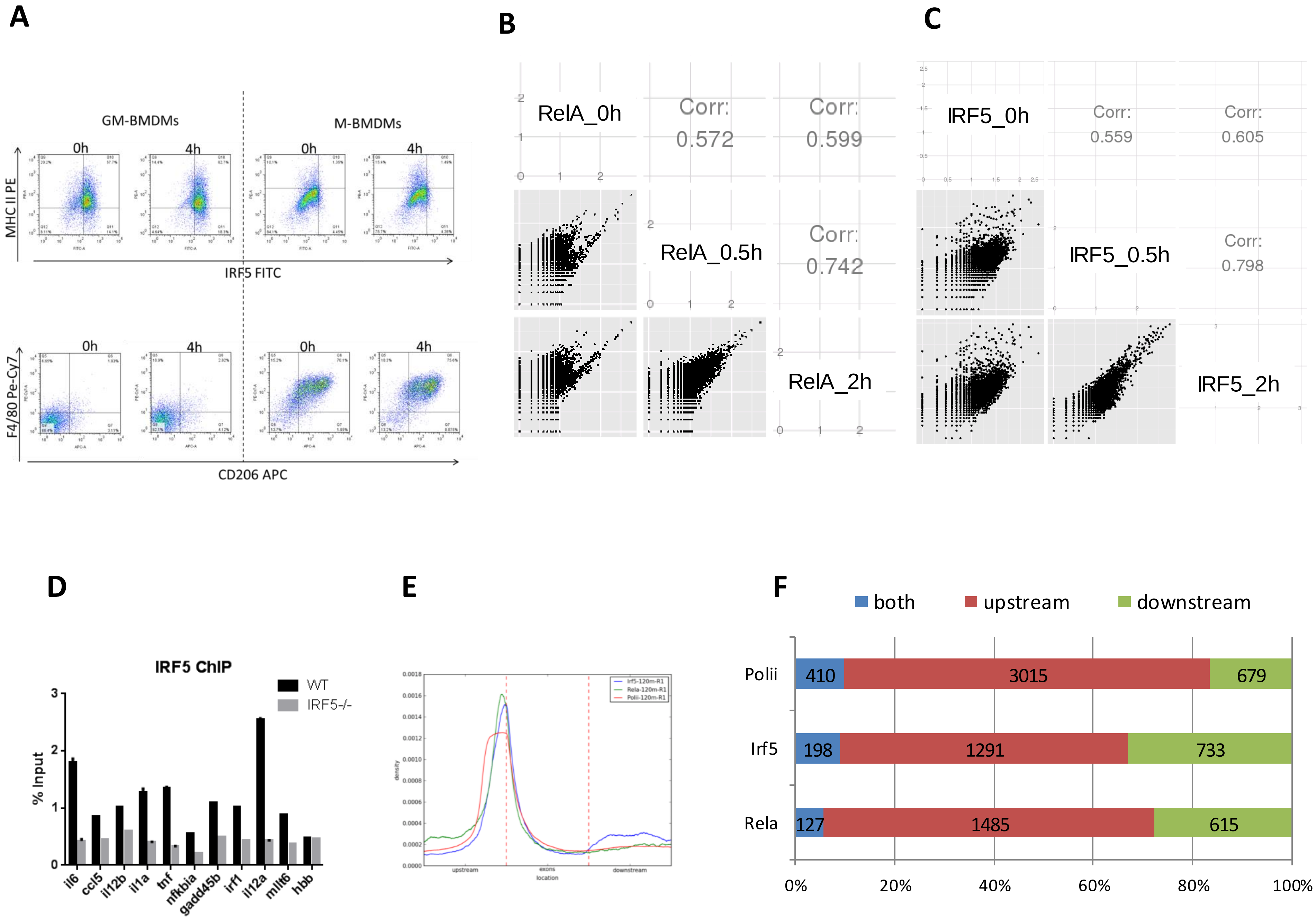

Supplementary Figure S1, related to Figure 1

A

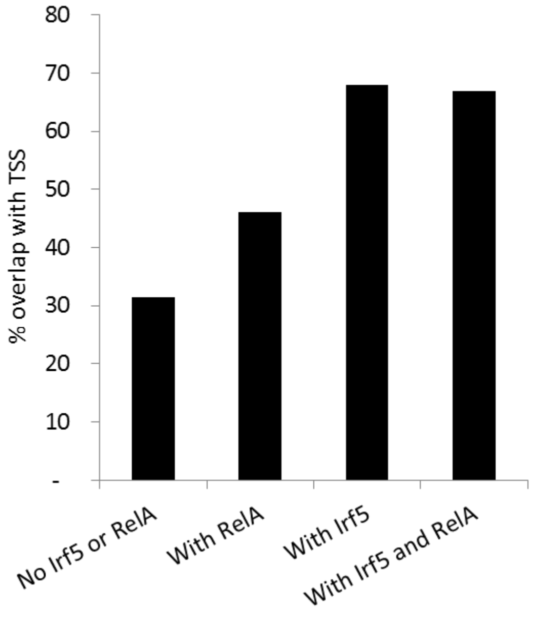

B

Promoter

Enhancer

RelA With Irf5

RelA Without Irf5

Irf5WithoutRelA

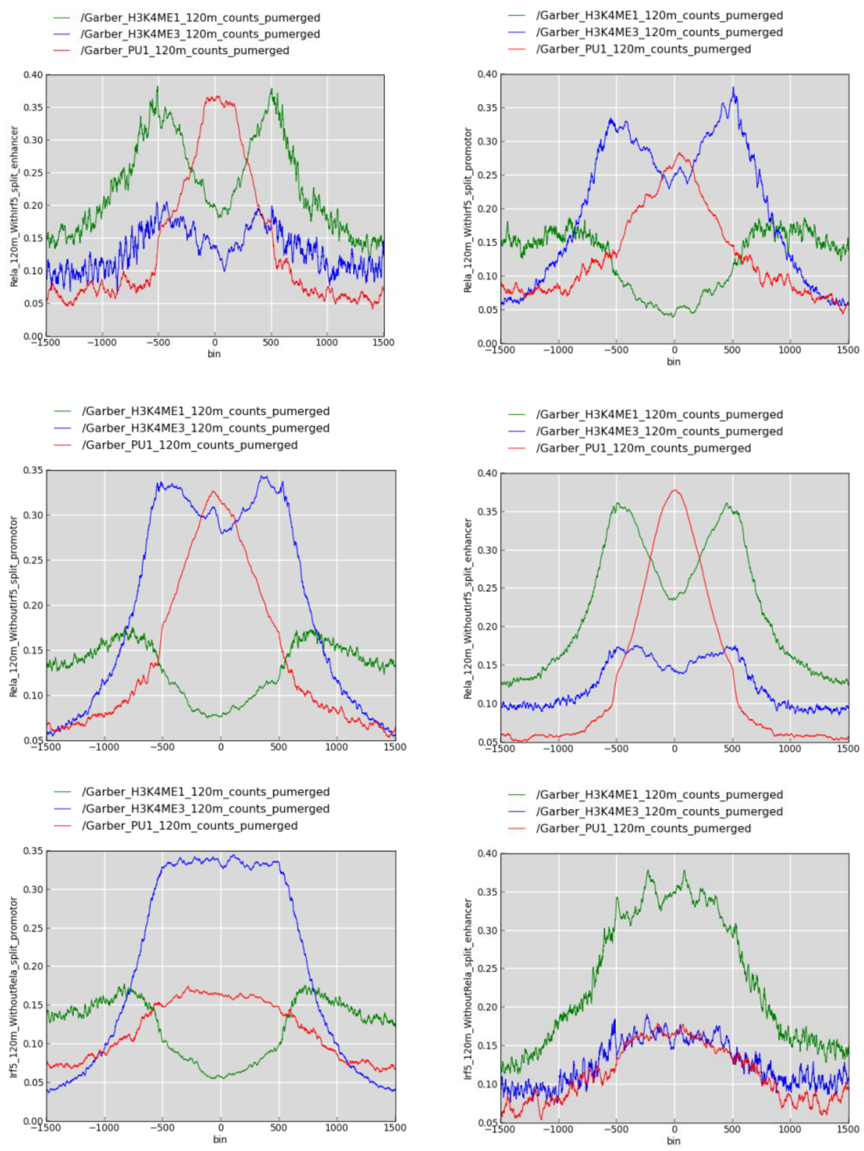

Supplementary Figure S2, related to Figure 2

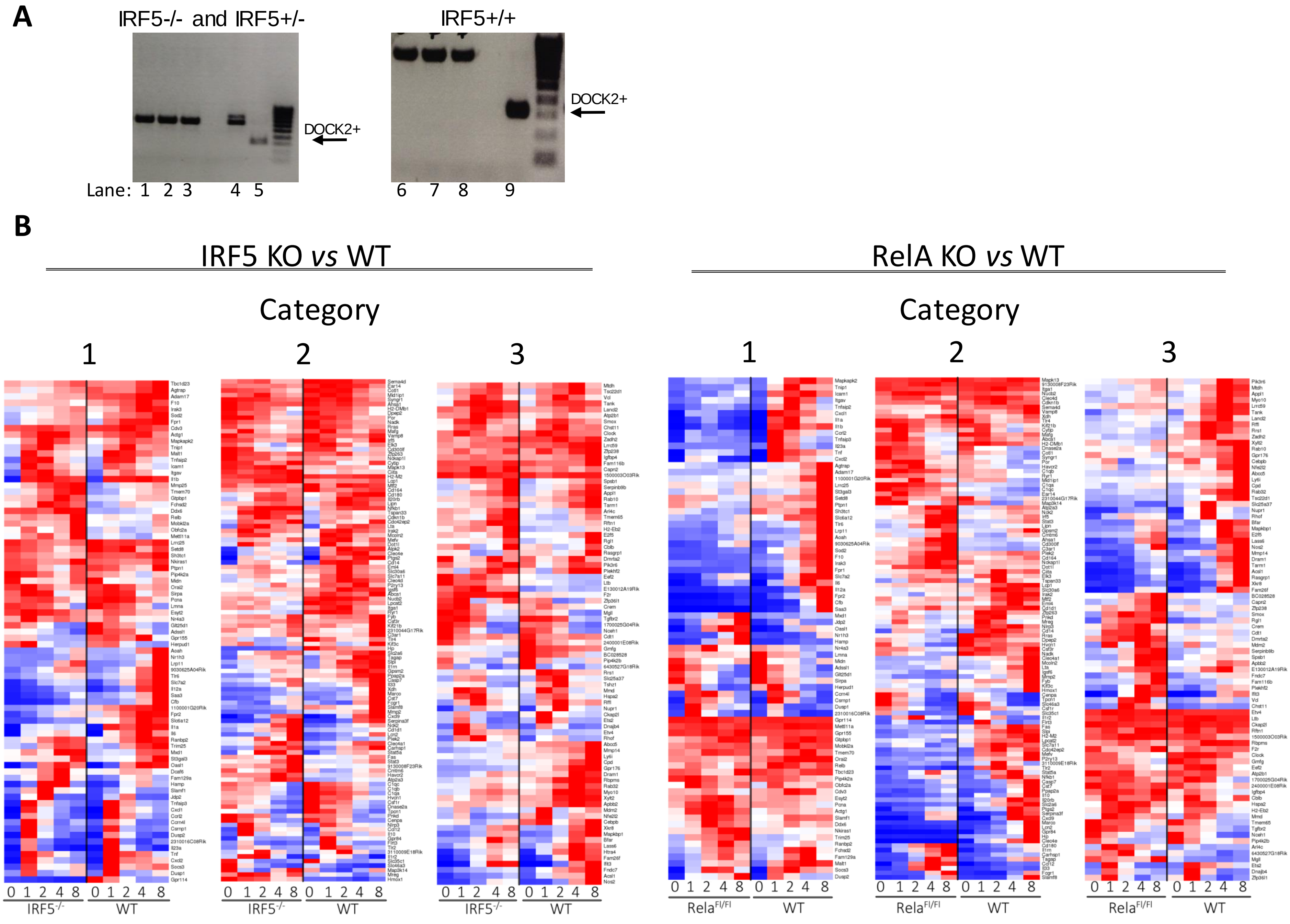

Supplementary Figure S3, related to Figure 3

Results combined from 2 arrays (IRF-3)

Correlation: 0.95

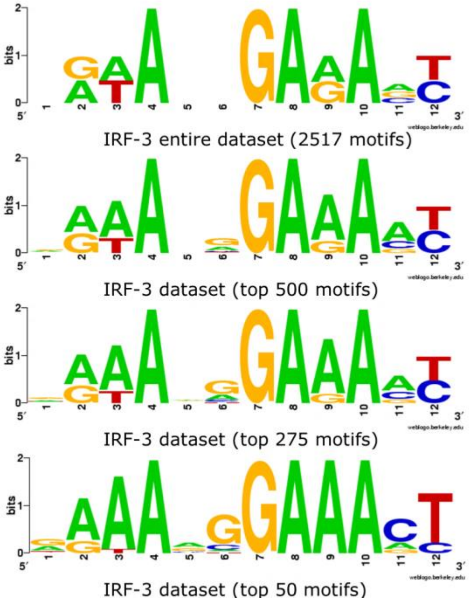

Results combined from 2 arrays (IRF-5)

Correlation: 0.96

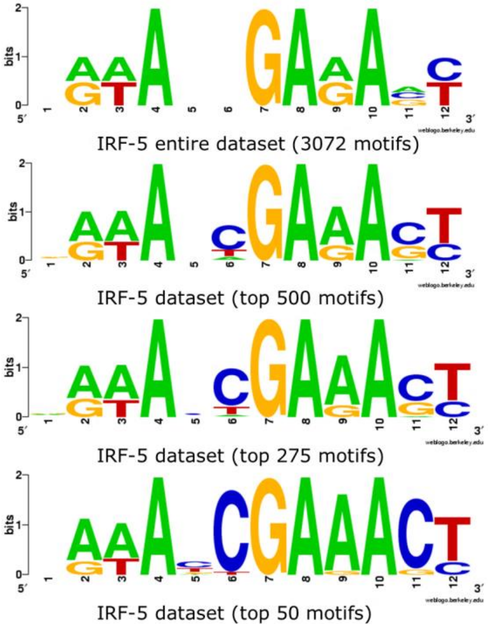

Supplementary Figure S4, related to Figure 4

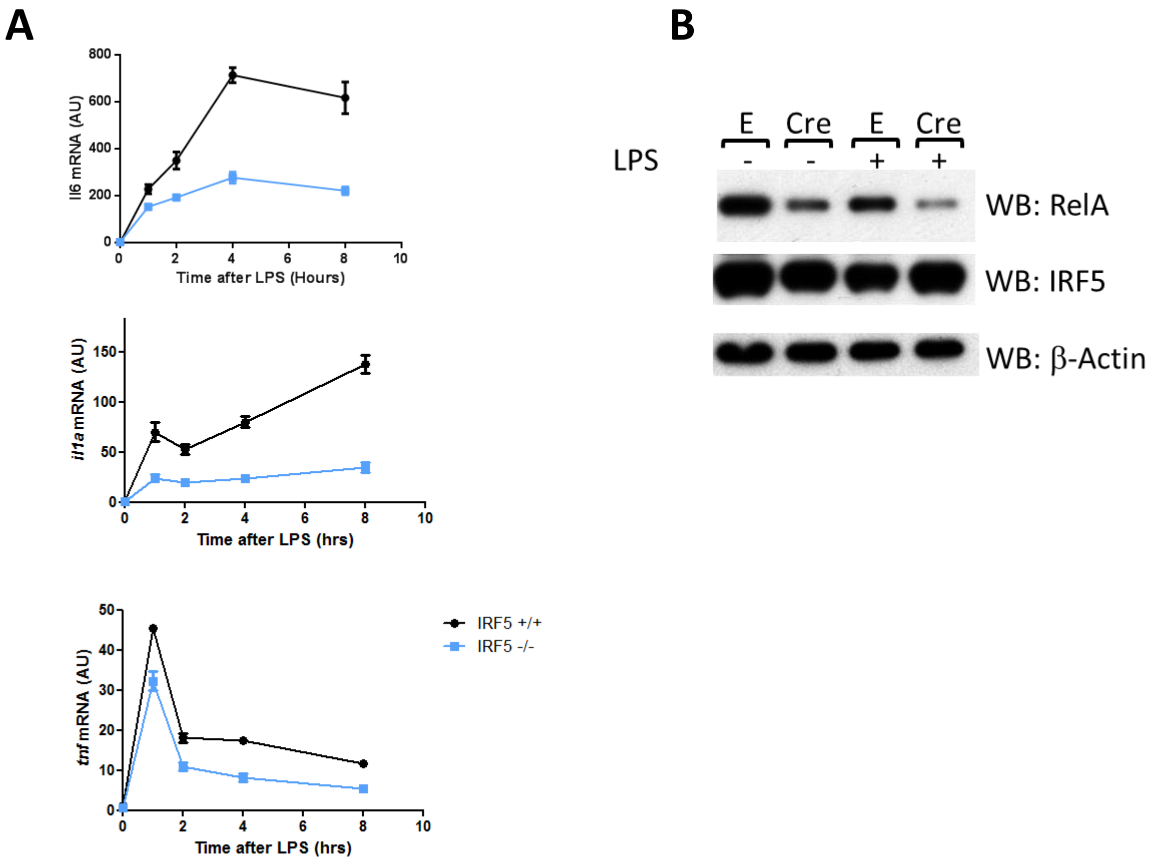

Supplementary Figure S5, related to Figure 5

Supplementary Table S1, related to Figure 1

A: number of sequences mapped to the genome

track	total	mapped	duplicates
Irf5/30'	22,921,738	17,486,718	4,352,213
Irf5/2h	11,006,735	7,928,653	1,174,331
Irf5/input	36,613,978	26,512,205	1,877,445
PolII/30'	2,372,589	1,437,092	108,247
PolII/2h	18,101,706	13,122,996	4,739,031
PolII/input	13,495,166	10,304,210	409,974
RelA/30'	8,722,397	5,235,902	334,544
RelA/2h	14,363,524	9,305,020	304,502
RelA/input	13,495,166	10,304,210	409,974

B: genome-wide distribution of mapped ChIP-Seq peaks

track	cds	utr	Up stream	Down stream	Intronic	Intergenic
IRF5/30'	57	58	119	194	1,583	2,103
IRF5/2h	281	508	490	480	1,264	970
PolII/2h	288	978	1,158	289	1,114	636
RelA/30'	146	584	1,073	241	1,965	1,684
RelA/2h	110	364	919	368	3,473	3,300

C: IRF5 ChIP-Seq interval nucleotide overlap with a genomic region 2h after LPS stimulation

annotation	observed	expected	CI95low	CI95high	stddev	fold	l2fold	p-value
intergenic	569,786	1,122,074	1,086,920	1,157,021	21116	0.5	-0.98	0.0001
intronic	854,072	1,209,453	1,174,133	1,244,637	21254	0.7	-0.50	0.0001
UTR3	64,216	42,083	34,133	50,265	4902	1.5	0.61	0.0001
CDS	147,997	59,050	52,683	65,651	3913	2.5	1.33	0.0001
upstream	329,704	105,740	92,234	119,578	8284	3.1	1.64	0.0001
downstream	330,675	96,689	83,460	110,237	8072	3.4	1.77	0.0001
UTR5	222,950	10,519	7,759	13,584	1768	21.2	4.41	0.0001

D: RelA ChIP-Seq interval nucleotide overlap with a genomic region 2h after LPS stimulation

annotation	observed	expected	CI95low	CI95high	stddev	fold	l2fold	pvalue
UTR3	58,864	73,239	63,548	83,260	5,984	0.8	-0.3	0.0063
CDS	82,724	101,229	93,341	109,260	4,846	0.8	-0.3	0.0001
intergen	1,730,230	2,017,149	1,975,502	2,059,215	25,629	0.9	-0.2	0.0001
intronic	1,903,324	2,126,694	2,085,354	2,169,435	25,622	0.9	-0.2	0.0001
downstream	197,311	168,714	153,064	184,640	9,539	1.2	0.2	0.0018
upstream	473,192	183,524	167,627	199,951	9,913	2.6	1.4	0.0001
UTR5	147,402	18,181	14,648	21,963	2,215	8.1	3.0	0.0001

Supplementary Table S2, related to Figure 2

A: IRF5 and RelA binding at promoters of strongly (>2-fold) up- and down-regulated genes. Differentially expressed genes are called at a false discovery rate of 1% and have a greater than two-fold change in expression.

**Category**	**Upregulated**	**Downregulated**	**Description**
IRF5+RelA	74	3	IRF5 and RelA present
RelA	65	12	Only RelA present
IRF5	53	30	Only IRF5 present

B: Fold enrichment of IRF5 and RelA ChIP-Seq peaks at chromatin marked regions

	**BMDM**		**BMDC**	
	**H3K4ME1**	**H3K4ME3**	**H3K4ME1**	**H3K4ME3**
**Irf5 (120')**	2.07	13.17	3.23	9.94
**RelA (120')**	8.20	7.53	5.48	6.31
**RelA and Irf5 (120')**	5.77	13.66	5.45	10.77

C: RelA:IRF5 binding at Pu.1 marked promoters and enhancers

	**H3K4ME1**	**H3K4ME3**
**All**	5.4	10.8
Pu.1	5.9	14.2
Without PU.1	1.5	5.6

A. Genes affected by IRF5 KO relative to WT following LPS stimulation

Category 1		Category 2		Category 3	
down	up	down	up	down	up
	Relb	***Slpi***	***Il1r2***	***Nfe2l2***	***Mgll***
Aoah	*Mobkl2a*	***Ppap2a***	*Tspan33*	***Acsl1***	*Etv4*
Il1a	*Slamf1*	***Csf1r***	*Cdkn1b*	***Ets2***	*Atp2b1*
Slc6a12	*Mmp25*	***Hp***	*Stat5a*	***Rasgrp1***	*Zfp36l1*
1100001G20Rik	*Tmem70*	***Mmp2***	*Mapk13*	***Gpr176***	*Ltb*
Fpr2	*Obfc2a*	***Gpr84***	*Atp2a3*	*Cpd*	*Rhof*
Adam17	*Fam129a*	***Hvcn1***	*Map3k14*	*Ly6i*	*Eef2*
Cxcl2	*Dcaf6*	*Elk3*	*Plek2*	*Lass6*	*Rftn1*
Cfb	*Pip4k2a*	*Abca1*	*Ciita*	*Apbb2*	*E130012A19Rik*
Saa3	*Mettl11a*	*Xdh*	*Cd1d1*	*Rbpms*	*F2r*
Il12a	*Fchsd2*	*Gpsm2*	*Slc46a3*	*Htra4*	*Tmem65*
Il23a	*Ddx6*	*Clec4d*	*Cmtm6*	*BC028528*	
Il6	*Gtpbp1*	*Ryr1*	*Mreg*	*Fndc7*	
Glt25d1		*Nucb2*	*9130008F23Rik*	*Tshz1*	
Gpr155		*Tpcn1*	*Nck2*	*Ckap2l*	
Socs3		*Alpk2*		*Gmfg*	
Gpr114		*Tlr4*		*Nos2*	
Adssl1		*Itga1*			
		Flrt3			
		Slamf8			

B. Genes affected by RelA KO relative to WT following LPS stimulation

Category 1		Category 2		Category 3	
down	up	down	up	down	up
Irak3	*Trim25*	***Slpi***	***Il1r2***	***Nfe2l2***	***Mgll***
Aoah	*Mxd1*	***Ppap2a***	*Dnase2a*	***Acsl1***	*Mmd*
Il6	*Nr1h3*	***Csf1r***	*Stat3*	***Ets2***	*Cdt1*
Il1a	*Ranbp2*	***Hp***	*Por*	***Rasgrp1***	*1500003O03Rik*
1100001G20Rik	*Esyt2*	***Mmp2***	*Mid1ip1*	***Gpr176***	*Fam116b*
Fpr2	*Nkiras1*	***Gpr84***	*Carhsp1*	*Mmp14*	*Igfbp4*
Adam17	*Ccrn4l*	***Hvcn1***	*Fcgr1*	*Pip4k2b*	*Mdm2*
Cxcl2	*Dusp1*	*Hmox1*	*Cd164*	*Rab32*	*Serpinb9b*
Cfb	*Pcna*	*Mcoln2*	*Havcr2*	*Dram1*	*Vcl*
Saa3	*Dusp2*	*H2-M2*	*Ahsa1*	*Rab10*	*Cblb*
Il12a	*Lmna*	*Il10*	*Cd180*	*Rffl*	*Rgl1*
Sod2	*Nr4a3*	*Kif3c*	*Syngr1*	*Xylt2*	*Capn2*
Mapkapk2	*Csrnp1*	*Sema4d*	*Nckap1l*	*Lrrc59*	*Smox*
Lrp11	*Jdp2*	*Lcp1*	*Lipn*	*Tsc22d1*	*Nceh1*
Tnfaip3	*Midn*	*Fyb*	*Il33*	*Mtdh*	*Dnajb4*
Tnip1	*Oasl1*	*Zfp263*	*Cytip*	*Myo10*	*2400001E08Rik*
Tnfaip2	*2310016C08Rik*	*Mefv*	*Il1rn*	*Bfar*	*Tgfbr2*
Tbc1d23	*Hamp*	*Slc30a6*	*Cenpa*	*Abcc5*	*1700025G04Rik*
Tnf	*Actg1*	*Lta*	*Irf5*	*E2f5*	*6430527G18Rik*
Itgav		*Fas*	*Cotl1*	*Clock*	*Chst11*
Il1b		*Casp7*	*Tagap*	*Nupr1*	*BC028528*
Ptpn1		*Pnkd*	*2310044G17Rik*	*Mapkbp1*	*Spsb1*
St3gal3		*3110009E18Rik*	*Ccl12*	*Slc25a37*	*Dmrta2*
Agtrap		*Marco*	*C1qa*	*Xkr8*	*Arl4c*
Cxcl1		*Lcn2*	*C1qc*	*Appl1*	*Plekhf2*
Slc6a12		*Slc7a11*	*C1qb*	*Fam26f*	*Hspa2*
F10		*Tlr2*	*C3ar1*	*Pik3r6*	*Zfp238*
Slc7a2		*Nfkb1*	*Kif21b*	*Zadh2*	*Crem*
Herpud1		*Csf3r*	*Cd300lf*	*Tarm1*	*H2-Eb2*
Malt1		*Nadk*	*Ear14*	*Cebpb*	*Ifit3*
Cdv3		*Mtf2*	*Dot1l*	*Rrs1*	
Sh3tc1		*Cxcl9*		*Lancl2*	
Icam1		*Clec4e*		*Tank*	
Sirpa		*Ptgs2*			
Orai2		*Eml4*			
Ccrl2		*Nlrp3*			
9030625A04Rik		*Lpcat2*			
Fpr1		*Igsf6*			
Setd8		*Slc2a6*			
Lrrc25		*P2ry13*			
Tlr6		*Rras*			
Relb		*Il20rb*			
		Cdc42ep2			
		Clec4a1			
		Slc35c1			
		Vamp8			
		Cd14			
		Mafg			
		Dpep2			
		Irak2			
		Serpina3f			
		Cst7			
		H2-DMb1			

Supplementary Table S3, related to Figure 3
